# Confinement controls the directional cell responses to fluid forces

**DOI:** 10.1016/j.celrep.2024.114692

**Published:** 2024-08-28

**Authors:** Farshad Amiri, Ayuba A. Akinpelu, William C. Keith, Farnaz Hemmati, Ravi S. Vaghasiya, Dylan Bowen, Razan S. Waliagha, Chuanyu Wang, Pengyu Chen, Amit K. Mitra, Yizeng Li, Panagiotis Mistriotis

**Affiliations:** 1 Department of Chemical Engineering, Auburn University, Auburn, AL 36849, USA; 2 Department of Drug Discovery and Development, Harrison College of Pharmacy, Auburn University, Auburn, AL 36849, USA; 3 Materials Research and Education Center, Auburn University, Auburn, AL 36849, USA; 4 Center for Pharmacogenomics and Single-Cell Omics (AUPharmGx), Harrison College of Pharmacy, Auburn University, Auburn, AL 36849, USA; 5 UAB O’Neal Comprehensive Cancer, University of Alabama at Birmingham School of Medicine, Birmingham, AL 35233, USA; 6 Department of Biomedical Engineering, Binghamton University, SUNY, Binghamton, NY 13902, USA; 7 Lead contact

## Abstract

Our understanding of how fluid forces influence cell migration in confining environments remains limited. By integrating microfluidics with live-cell imaging, we demonstrate that cells in tightly—but not moderately—confined spaces reverse direction and move upstream upon exposure to fluid forces. This fluid force-induced directional change occurs less frequently when cells display diminished mechanosensitivity, experience elevated hydraulic resistance, or sense a chemical gradient. Cell reversal requires actin polymerization to the new cell front, as shown mathematically and experimentally. Actin polymerization is necessary for the fluid force-induced activation of NHE1, which cooperates with calcium to induce upstream migration. Calcium levels increase downstream, mirroring the subcellular distribution of myosin IIA, whose activation enhances upstream migration. Reduced lamin A/C levels promote downstream migration of metastatic tumor cells by preventing cell polarity establishment and intracellular calcium rise. This mechanism could allow cancer cells to evade high-pressure environments, such as the primary tumor.

## In brief

Amiri et al. report that fluid forces cause upstream migration of confined cells via mechanisms involving the cytoskeleton, nucleus, and ion channels. This migration is less likely under conditions of chemotaxis or high hydraulic resistance. Metastatic cells with low levels of lamin A/C display reduced mechanosensitivity, resulting in downstream migration.

## Graphical Abstract

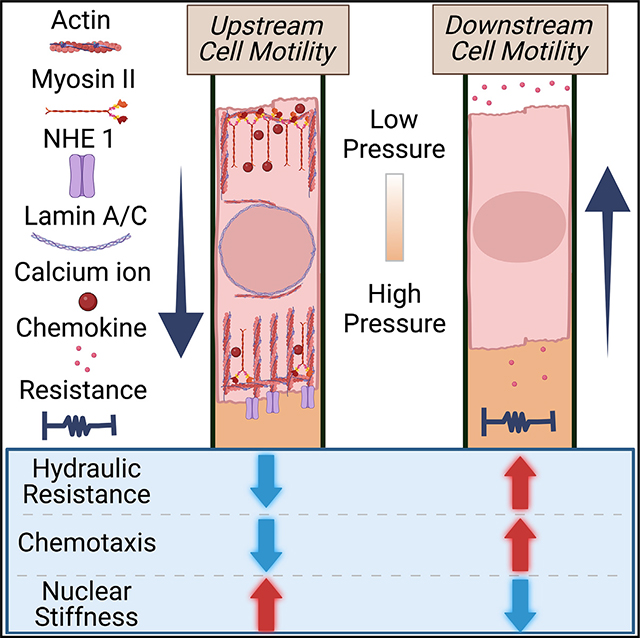

## INTRODUCTION

Cell migration is a complex biological process that controls a wide range of (patho)physiological events, including tissue regeneration and cancer metastasis. Although the prevailing understanding is that the physical cues of the cell microenvironment (e.g., viscosity,^1–3^ confinement,^4–11^ viscoelasticity,^12^ plasticity,^13^ stiffness,^14–18^ hydraulic resistance,^19–22^ and pressure^23^) influence cell migratory behavior, little is known about how migrating cells sense and respond to asymmetric physical signals, including pressure differentials (ΔP).

Osmotic and hydrostatic ΔP govern fluid flow *in vivo*, including blood and interstitial flow. The interstitial fluid velocity, typically between 0.1 and 10 μm/s,^24^ is considerably slower than that of blood, which ranges from several hundred micrometers to millimeters per second in microvessels.^25^ Pathological conditions like cancer can dramatically increase interstitial flow by a factor of 3–5^26^ due to the elevated interstitial fluid pressure (IFP) within tumors relative to the adjacent healthy tissue, which triggers outward convection.^27,28^ Pressure-generated flow fields exert fluid forces (i.e., shear and/or pressure forces) on migrating cells in the direction of flow.

To date, there is limited understanding of the factors and mechanisms that govern directional cell responses to fluid forces. Prior work using mesenchymal-like cells in 3D hydrogels has shown distinct behavior of cell subpopulations in response to flow; while some cells move upstream (i.e., against the flow), others migrate downstream (i.e., with the flow).^29,30^ Interestingly, dense cultures suppress downstream migration while triggering cell movement in the opposite direction.^29,31,32^ Because changes in cell density and variations in pore size within hydrogels influence the degree of cell confinement, we hypothesized that cells exhibit distinct directional responses to fluid forces in moderately versus tightly confined microenvironments. To test this, we opted for microchannel devices^6,33^ over 3D hydrogels because the former allow for precise control of spatial dimensions independent of other physical parameters, such as stiffness, enabling us to isolate the effects of confinement on cell migration. By generating hydrostatic pressure gradients across microchannels, whose dimensions recapitulated the size of pores or channel-like tracks encountered by migrating cells *in vivo*,^34,35^ we demonstrate that increased confinement prompts cell movement to regions of higher pressure. Our findings may have broader implications for cancer metastasis, as they reveal mechanisms that could prevent cancer cells from escaping high-pressure environments, such as the primary tumor.

## RESULTS

### Tight confinement triggers upstream cell migration

**Figure 1.**
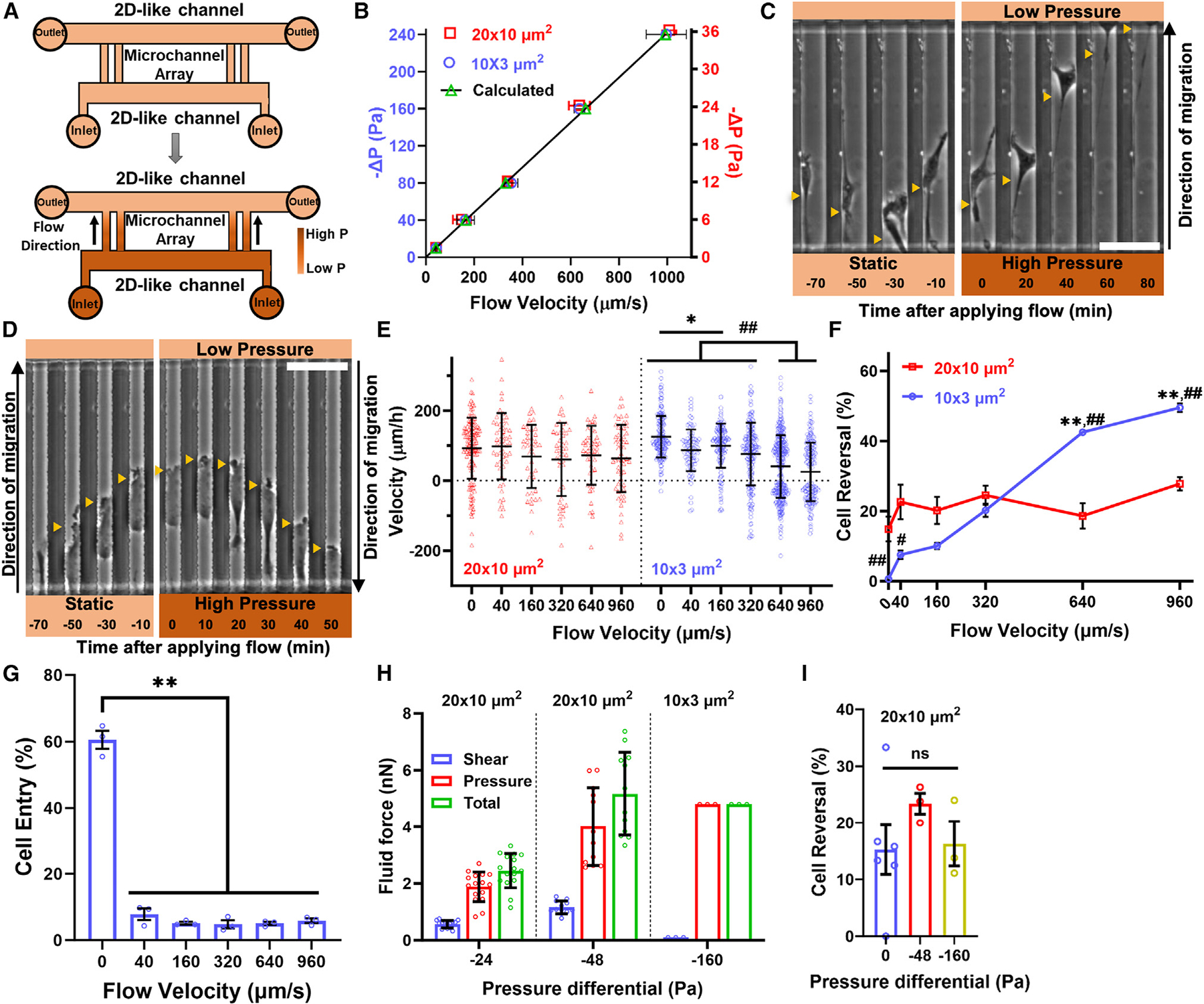
Upstream cell migration in tight confinement (A) Schematic of our assay that assesses cell migration in moderately (20 × 10 μm^2^) or tightly (10 × 3 μm^2^) confined microchannels under static (top) or flow (bottom) conditions. (B) Calculated and experimentally measured pressure-driven flow within moderately and tightly confined microchannels. ≥40 beads per experiment, 3 experiments. (C and D) Image sequence of HT-1080 cells migrating in (C) moderately or (D) tightly confined microchannels before and after initiation of flow (640 μm/s in empty microchannels). Yellow arrowheads indicate cell position. Scale bar, 50 μm. (E) HT-1080 cell velocity in moderately and tightly confined microchannels under static (0 μm/s) and various flow conditions. *n* ≥ 41 cells; 3 experiments; *p < 0.05, ##*p* < 0.01. (F) The percentage of HT-1080 cells that reverse their direction in moderately and tightly confined microchannels under static (0 μm/s) and various flow conditions. ≥10 cells per experiment; ≥3 experiments; **p < 0.01 relative to 0, 40, and 160 μm/s (10 × 3 μm^2^); #*p* < 0.05 and ##*p* < 0.01 relative to 320 μm/s (10 × 3 μm^2^). (G) The percentage of HT-1080 cells that enter tightly confined microchannels under static (0 μm/s) and various flow conditions. ≥134 cells per experiment, ≥3 experiments, **p < 0.01. (H) Shear, pressure, and total drag exerted on moderately and tightly confined cells at different ΔP. *n* ≥ 12 cells. (I) The percentage of HT-1080 cells that reverse their direction in moderately confined microchannels following exposure to different ΔP. ns, not significant; ≥18 cells per experiment; ≥3 experiments. Statistical tests: one-way ANOVA (E, G, and I) and two-way ANOVA (F) followed by Tukey’s multiple-comparisons post hoc test. Values represent mean ± SD (E and H) or mean ± SEM (B, F, G, and I). See also Figure S1.

To study fluid force-dependent migration in confinement, we employed microfluidic devices containing moderately (width [*W*] × height [*H*] = 20 × 10 μm^2^) or tightly (*W* × *H* = 10 × 3 μm^2^) confined collagen I-coated microchannels of constant length (length [*L*] = 200 μm) (Figure 1A). These microchannels were aligned in a parallel arrangement and positioned between two larger, 2D-like channels that served as reservoirs for cells and culture medium (Figure 1A). By calculating the resistance of each microfluidic circuit (Figure S1A), we determined the ΔP required to generate fluid velocities within microchannels, ranging from 0 to ~960 μm/s (Figure 1B). To achieve these velocities, the ΔP ranged from 0 to −36 Pa and from 0 to −240 Pa for moderately and tightly confined microchannels, respectively (Figure 1B). We validated our velocity projections by introducing microbeads into our devices and increasing the height of the medium in the inlet relative to the outlet wells, thereby generating a wide range of negative ΔP (Figures 1A and 1B). Moreover, we measured identical flow velocities in microchannels located at the center versus the edge of the device (Figure S1B) due to the negligible pressure drop (<1 Pa) occurring in the lowermost 2D-like channel.

Next, we examined how flow influenced confined migration. HT-1080 fibrosarcoma cells were seeded in the lowermost, 2D-like channel and allowed to migrate through moderately or tightly confined microchannels in an isobaric environment (ΔP = 0) toward a chemoattractant (10% [v/v] fetal bovine serum [FBS]) placed in the uppermost 2D-like channel (Figures 1A, 1C, and 1D). The chemoattractant was used to facilitate channel entry and was removed in later phases of the experiment. At t = 0, we initiated directional fluid flow from the lowermost to the uppermost 2D-like channels by generating −ΔP, as described above. While HT-1080 cell migration in moderately confined channels remained largely unaffected by variations in fluid flow, the velocity of tightly confined cells decreased progressively as the fluid flow increased (Figures 1C–1E; Videos S1 and S2) due to the rapid reversal (within ~30–40 min) in the migration direction observed in a fraction of HT-1080 cells, as indicated by the negative cell velocity values (Figures 1D–1F). This confinement-induced direction change led to migration toward higher-pressure regions (Figure 1D). These findings held true for MDA-MB-231 breast cancer cells and even non-cancerous cells, such as human bone marrow mesenchymal stem cells (MSCs) and adipose-derived MSCs (AD-MSCs) (Figures S1C–S1H). Interestingly, AD-MSCs displayed increased migration with the flow in moderate confinement (Figure S1H). Furthermore, cells residing adjacent to the microchannel entrances exhibited an extremely low tendency to enter tight confinement and consequently migrate downstream, as shown using various cell types (Figures 1G and S1I). This was observed even at lower fluid velocities (40 μm/s) (Figure 1G).

Next, we focused on the flow of 640 μm/s, established by applying a −ΔP of 24 Pa and 160 Pa across moderately and tightly confined channels, respectively (Figure 1B). These flow conditions induced upstream migration in ~40% of tightly confined HT-1080 cells without impacting the motility of moderately confined cells (Figures 1E and 1F). Using microbeads, we observed that, in microchannels containing cells, flow either came to a complete halt, as observed in tightly confined scenarios, or was reduced, as seen within moderately confined conditions (Figure S1J). However, microchannels lacking cells, a common occurrence in our devices due to the low seeding cell density, maintained a flow velocity of 640 μm/s, similar to that of cell-free devices (Figures 1B and S1J).

The absence of fluid flow in tightly confined microchannels filled with HT-1080 cells indicates a lack of shear drag. Consequently, the total fluid forces acting on these cells are attributed to pressure drag, which can be calculated by multiplying the pressure drop across the microchannel (160 Pa) with the microchannel perpendicular cross-sectional area (30 μm^2^). This force amounts to 4.8 nN (Figure 1H). In comparison, moderately confined HT-1080 cells experience both shear and pressure drag due to the presence of a non-zero fluid velocity in these microchannels. Approximating the cell spreading area as a rectangle with a width equal to the channel width (*W* = 20 μm) and calculating the volume flow rate from the measured average bead velocity allows estimation of the viscous shear stress on the cell as well as the pressure drop across the cell. These values are then converted into shear and pressure drag by multiplying them with the longitudinal (*W* × *L*_*cell*_) and perpendicular (*H*_*cell*_ × *W*) cross-sectional areas of individual cells, respectively. The average pressure drag on moderately confined HT-1080 cells surpasses shear drag by 3-fold (1.9 ± 0.5 nN vs. 0.6 ± 0.1 nN) (Figure 1H). Moreover, the total fluid force on these cells is estimated to be 2.5 ± 0.6 nN, representing half the force experienced by tightly confined HT-1080 cells under the same flow conditions (Figure 1H).

To rule out the possibility that the distinct migratory behavior of tightly and moderately confined cells under flow (Figures 1E and 1F) was due to differences in the magnitude of the total fluid forces they experienced, we established flow conditions that generated comparable drag forces for both cell populations (~5 nN). We found that a −ΔP of 48 Pa produced a flow of ~1,200 μm/s in empty, moderately confined channels and yielded an ~4-nN pressure force and an ~1.2 nN shear force on moderately confined cells (Figure 1H). These flow conditions failed to trigger upstream migration in moderate confinement when compared to static conditions (Figure 1I). Similar observations were made for −ΔP of 160 Pa (Figures 1I and S1K), expected to exert an ~16.5-nN fluid force on moderately confined cells. In sum, drag forces, ranging from 0–16.5 nN, fail to induce migration against the flow in moderate confinement. However, upstream migration can be observed in tight confinement even at a −ΔP as low as 10 Pa, which results in a 0.3-nN fluid force.

### Chemotaxis and elevated hydraulic resistance suppress confinement-induced upstream migration

**Figure 2.**
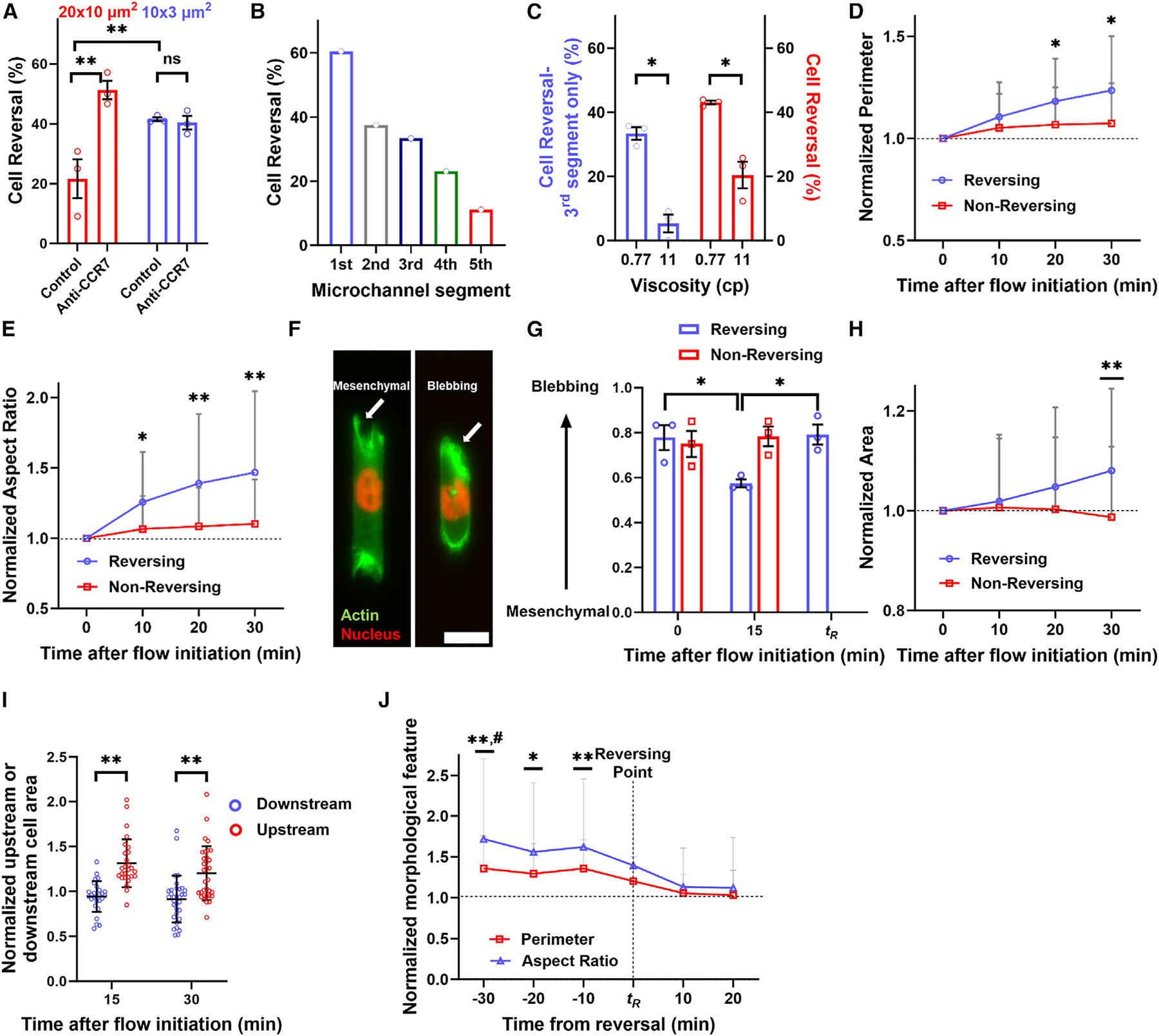
Chemotactic signals and hydraulic resistance regulate fluid force-induced cell reversal (A) The percentage of HT-1080 cells treated with isotype control or anti-CCR7 antibody that reverse their direction in moderately and tightly confined microchannels following exposure to an ~5-nN fluid force. ≥9 cells per experiment, ≥3 experiments, **p < 0.01. (B) The percentage of HT-1080 cells that reverse their direction in different microchannel segments following exposure to a 4.8-nN fluid force. ≥13 cells pooled from 6 experiments. (C) The percentage of HT-1080 cells that reverse their direction in tightly confined microchannels following exposure to a 4.8-nN fluid force. The cell culture media used have viscosities of 0.77 cP or 11 cP. ≥14 cells per experiment, 3 experiments, *p < 0.05. (D and E) Normalized (D) perimeter and (E) aspect ratio of reversing and non-reversing, tightly confined HT-1080 cells following exposure to a 4.8 nN fluid force. Values are normalized to the flow initiation time point (t = 0). *n* ≥ 88 cells; ≥6 experiments; *p < 0.05, **p < 0.01 reversing relative to non-reversing. (F) Representative images of LifeAct-GFP/H2B-mCherry-labeled, tightly confined HT-1080 cells exhibiting a mesenchymal or bleb-based migration phenotype. Scale bar, 20 μm. (G) Migration phenotype of reversing and non-reversing LifeAct-GFP/H2B-mCherry-labeled, tightly confined HT-1080 cells immediately (t = 0) and 15 min after flow initiation (4.8 nN fluid force) as well as at the cell reversal time point (*t*_R_). ≥19 cells per experiment, 3 experiments, *p < 0.05. (H) Normalized area of reversing and non-reversing tightly confined HT-1080 cells following exposure to a 4.8-nN fluid force. *n* ≥ 97 cells, ≥7 experiments, **p < 0.01 reversing relative to non-reversing. (I) Normalized upstream or downstream area of reversing LifeAct-GFP/H2B-mCherry-labeled HT-1080 cells in tightly confined microchannels 15 and 30 min after flow initiation (4.8 nN fluid force). Values are normalized to the upstream or downstream cell area immediately after flow initiation. *n* ≥ 27 cells, ≥3 experiments, **p < 0.01. (J) Normalized perimeter and aspect ratio of reversing HT-1080 cells in tightly confined microchannels before and after the *t*_R_ (4.8 nN fluid force). *n* ≥ 88 cells, ≥6 experiments; *p < 0.05 perimeter at t = −20 min relative to perimeter at t = 10 and t = 20 min, **p < 0.01 perimeter at t = −30 or −10 min relative to perimeter at t = 10 and t = 20 min, #*p* < 0.05 aspect ratio at t = −30 min relative to aspect ratio at t = 10 and t = 20 min. Statistical tests: paired Student’s t test (C), one-way ANOVA (G and J), and two-way ANOVA (A, D, E, H, and I) followed by Tukey’s multiple-comparisons post hoc test. Values represent mean ± SD (D, E, and H–J) or mean ± SEM (A, C, and G). See also Figure S2.

Next, we investigated the factors influencing fluid force-induced directional change in tight confinement. Cell reversal, triggered by a 4.8-nN fluid force, was independent of cell density in the lowermost 2D-like channels (Figures S2A and S2B) or the precise placement of the cell population within the device (Figures S2C and S2D). The use of FBS as a chemoattractant during the initial migration phase, prior to the flow initiation, had no impact on fluid force-induced cell reversal (Figure S2E). However, maintaining FBS within the uppermost channel throughout the entire experiment reduced the fraction of cells migrating upstream (Figure S2E). Because autocrine-secreted C-C chemokine receptor type 7 (CCR7) ligands form gradients under flow conditions, facilitating downstream migration in 3D hydrogels,^36^ we investigated the role of CCR7 in the rheotactic behavior of confined cells. Antibody-mediated blocking of CCR7 failed to alter the fraction of tightly confined cells moving upstream (Figure 2A), presumably because the absence of flow in tightly confined channels filled with cells prevented the formation of pericellular gradients of CCR7 ligands. Intriguingly, this intervention triggered upstream cell migration in moderate confinement (Figure 2A). These data indicate that downstream accumulation of chemotactic signals can, at least in part, counteract the effects of fluid forces on cell reversal.

To migrate upstream, tightly confined cells must overcome the microchannel’s hydraulic resistance, which, for rectangular channels, is proportional to the channel length.^21^ We thus hypothesized that it would be progressively more difficult for cells to reverse their direction as their distance from the channel entrance increased. To test this, we divided the 200-μm-long microchannels into five equal segments (Figure S2F) and assessed cell reversal in each segment after exposure to a 4.8-nN fluid force. Cells were classified as “reversing” if they exhibited a migration direction change, resulting in upstream movement, and as “non-reversing” if they maintained their direction, leading to downstream migration. HT-1080 cells had a higher likelihood of migrating toward low-pressure environments when positioned within the latter half of the microchannel, whereas those situated at the beginning of the microchannel were more prone to move upstream (Figure 2B). To further confirm that elevated hydraulic resistance hindered reversal of tightly confined cells, we increased the viscosity of the cell culture medium from ~0.8 cP to 11 cP at 37°C by adding methylcellulose and, thus, ensuring no alteration to the medium’s osmolarity.^1^ This increase in extracellular viscosity elevated hydraulic resistance by a factor of 14 since hydraulic resistance is directly proportional to fluid viscosity.^21^ Increased viscosity markedly suppressed fluid force-induced cell reversal, as shown for cells randomly positioned in the microchannels and for those in the third microchannel segment (Figure 2C).

Next, we compared the phenotype and morphology of reversing and non-reversing HT-1080 cells in tight confinement. Immediately after exposure to a 4.8-nN fluid force, both populations displayed comparable features, such as area, perimeter, and aspect ratio (Figures S2G–S2I). Interestingly, this fluid force induced a rapid increase in the perimeter and aspect ratio of reversing cells over time (Figures 1D, 2D, and 2E), indicating a shift toward a more elongated morphology. Cell elongation was accompanied by the transition of a subset of cells from a bleb-based migration phenotype, marked by plasma membrane blebbing, to a mesenchymal migration mode characterized by prominent actin-rich protrusions (Figures 2F and 2G). Moreover, we observed a modest (~8%) but significant increase in the overall cell area (Figure 2H). Cell expansion occurred due to growth in the upstream, but not downstream, direction (Figures 1D and 2I). Approximately 10 min before the cell reversal time point (*t*_R_), the upstream protrusions started to retract, resulting in a reduction in aspect ratio and perimeter (Figure 2J). This coincided with upstream cell movement and the reappearance of membrane blebs (Figure 2G). Non-reversing cells maintained their migration phenotype and displayed no morphological changes under the same flow conditions (Figures 2D, 2E, 2G, and 2H). In sum, fluid force-induced cell reversal, initially marked by the formation of protrusions extending upstream, is accompanied by substantial morphological and phenotypic changes not detected in non-reversing cells.

### Fluid forces alter the localization of actin and myosin II to promote cell reversal

**Figure 3.**
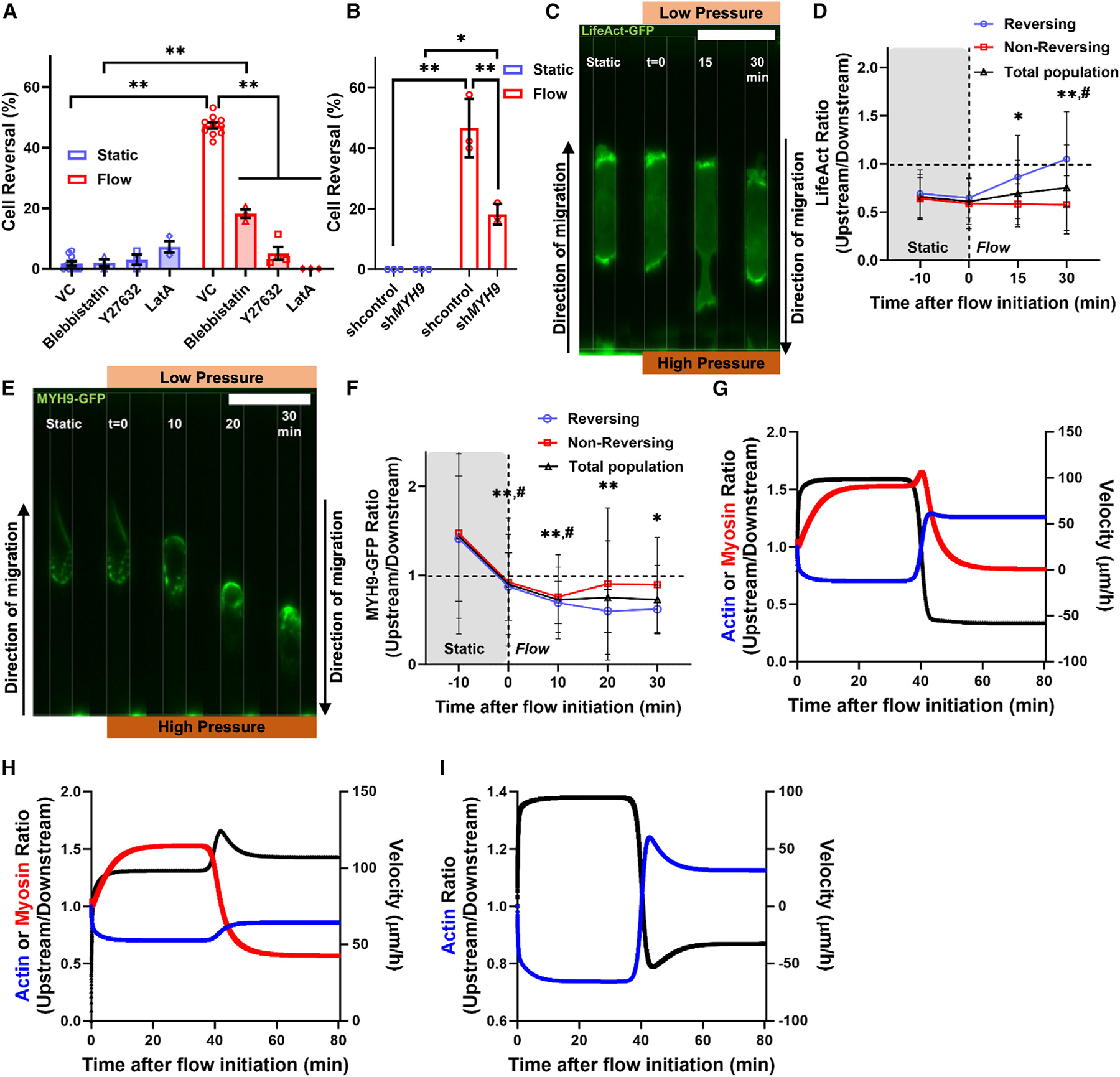
Fluid forces induce actin and myosin II redistribution in tightly confined cells (A and B) The percentage of (A) vehicle control (VC)-, blebbistatin-, Y27632-, and latrunculin A (LatA)-treated HT-1080 cells and (B) shcontrol and sh*MYH9* HT-1080 cells that reverse their direction in tight confinement under static conditions and following exposure to a 4.8-nN fluid force. (A) ≥26 cells per experiment, 3 experiments, **p < 0.01. (B) ≥20 cells per experiment, 3 experiments; *p < 0.05, **p < 0.01. (C) Image sequence of a LifeAct-GFP-labeled HT-1080 cell before and after flow initiation (4.8 nN fluid force). Scale bar, 50 μm. (D) Upstream-to-downstream ratio of LifeAct-GFP signal in reversing, non-reversing, and the total population of HT-1080 cells before and after flow initiation (4.8-nN fluid force). *n* = 59 cells, 3 experiments; #*p* < 0.05 reversing at t = 30 min relative to reversing t = −10, 0, and 15 min, *p < 0.05 reversing at t = 15 min relative to non-reversing at t = 15 min, **p < 0.01 reversing at t = 30 min relative to non-reversing at t = 30 min. (E) Image sequence of an MYH9-GFP-labeled HT-1080 cell before and after flow initiation (4.8-nN fluid force). Scale bar, 50 μm. (F) Upstream-to-downstream ratio of MYH9-GFP signal in reversing, non-reversing, and the total population of HT-1080 cells before and after flow initiation (4.8-nN fluid force). *n* = 40 cells, 3 experiments; *p < 0.05 reversing at t = 30 min relative to reversing at t = −10 min, **p < 0.01 reversing at t = 0, 10, or 20 min relative to reversing at t = −10 min, #*p* < 0.05 non-reversing at t = 0 or 10 min relative to non-reversing at t = −10 min. (G) A model showing cell velocity and the concentration ratios of F-actin and myosin as a function of time. (H) A model showing cell velocity in the absence of a fluid force-induced actin redistribution. Here we used $J_{c,\text{active}}^{b}=10\;mM\;\mu \;m/s$ after the pressure drop to induce myosin repolarization. (I) A model without myosin, otherwise the same as (G). Statistical tests: two-way ANOVA (A, B, D, and F) followed by Tukey’s multiple-comparisons post hoc test. Values represent mean ± SD (D and F) or mean ± SEM (A and B). See also Figure S3.

Changes in migration phenotype and morphology suggest that reversing cells undergo cytoskeletal remodeling, potentially involving actin polymerization and myosin II contractility. Given the established roles of actin and myosin II in cell migration,^37,38^ we investigated their contributions to upstream cell motility. Inhibition of actin polymerization via treatment of HT-1080 cells with latrunculin A (LatA; 2 μM) abolished fluid force (4.8 nN)-induced directional change in tight confinement, promoting exclusively downstream migration (Figures 3A and S3A). Similar results were obtained using the Rho-associated kinase inhibitor Y27632 (10 μM), which blocked both actomyosin contractility and actin stress fibers (Figures 3A and S3A). Moreover, inhibition of myosin II via short hairpin RNA (shRNA) knockdown (KD) of myosin IIA (sh*MYH9*) (Figure S3B) or the myosin II ATPase cycle inhibitor blebbistatin (20 μM) reduced, but did not prevent, upstream cell motility (Figures 3A, 3B, and S3A). Under static conditions, none of these interventions had any discernible impact on migration direction (Figures 3A and 3B). However, under the same conditions, blebbistatin or Y27632 treatment suppressed cell entry into tightly confined microchannels (Figure S3C). Consistent with their role in promoting downstream migration (Figures 3A and S3A), blebbistatin or Y27632 enhanced channel entry under flow conditions (Figure S3C).

To monitor the dynamics of actin and myosin IIA, we employed the probes LifeAct-green fluorescent protein (GFP) and MYH9-GFP. Consistent with prior findings,^5,39^ cells migrating under tightly confined, static conditions displayed actin predominantly at the front and myosin IIA at the rear (Figures 3C–3F). A 4.8-nN fluid force induced a rapid redistribution of actin and myosin IIA in reversing cells that coincided with a change in migration direction (Figures 3C–3F). Actin depolymerized on the cell side exposed to lower pressure and polymerized upstream, while myosin IIA levels declined on the high-pressure side and accumulated downstream (Figures 3C–3F). In non-reversing cells, we observed a downstream polarization of myosin IIA with no corresponding change in actin (Figures 3D and 3F), suggesting that myosin II redistribution was insufficient to initiate cell reversal in the absence of actin reorganization. Moderately confined cells failed to redistribute actin and myosin in response to fluid forces (Figures S3D and S3E).

Because it is experimentally challenging to selectively inhibit cytoskeletal elements at the cell front or rear, we developed a biophysics-based mathematical model to investigate the contributions of cytoskeletal reorganization to cell reversal in tightly confined microchannels. The model encompasses pressure gradients across tightly confined microchannels, fluid fluxes across the plasma membrane, solute fluxes from ion transporters and across passive channels, as well as actin and myosin dynamics. The fluid, solutes, actin, and myosin are tightly coupled through force balance and convection interaction. Such a coupled model is able to determine the driving mechanism of cell migration and reversal. In agreement with experiments (Figures 3C–3F), our model showed that actin and myosin polarized at the cell front and rear, respectively, in forward-moving, tightly confined cells (Figure 3G; Video S3). Migration direction reversal redistributed actin and myosin to the new cell front and rear, respectively (Figure 3G; Video S3), consistent with experimental observations (Figures 3C–3F). Our model predicted that failure of actin to repolarize prevented migration direction reversal even when myosin accumulated downstream (Figure 3H). Actin repolarization to the new cell front triggered upstream migration even in the absence of myosin II, albeit at a lower velocity (Figure 3I). Collectively, experiments and modeling suggest that upstream polymerization of actin is required for migration direction reversal. Myosin II facilitates this process, presumably by generating contractile forces that mediate retraction of the new cell rear.

### Intracellular calcium and NHE1 act in concert to induce cell reversal in tight confinement

**Figure 4.**
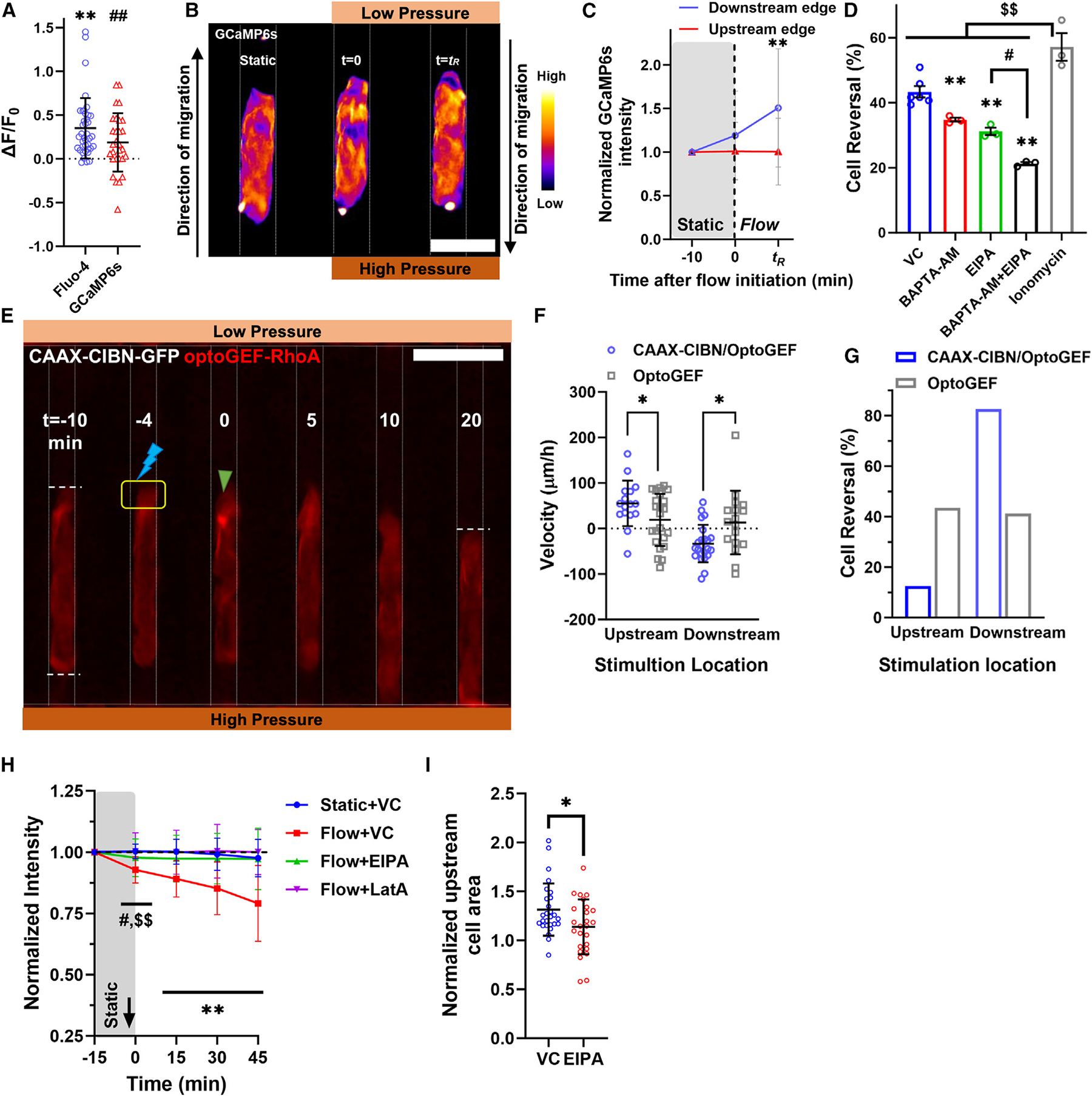
Confinement-induced upstream migration is controlled by NHE1 and calcium (A) Quantification of Fluo-4 Direct fluorescence and GCaMP6s intensity in HT-1080 cells in tightly confined microchannels. ΔF = F – F_0_, F = fluorescence immediately after exposure to a 4.8-nN fluid force, F_0_ = mean fluorescence intensity under static conditions. *n* ≥ 30 cells, 3 experiments; **p < 0.01 relative to the static condition for Fluo-4 Direct, ##*p* < 0.01 relative to the static condition for GCaMP6s. (B) Image sequence showing GCaMP6s intensity in an HT-1080 cell before and immediately after flow initiation as well as at the *t*_R_ (4.8-nN fluid force). Scale bar, 20 μm. (C) Normalized upstream and downstream GCaMP6s intensity in HT-1080 cells before and immediately after flow initiation (4.8-nN fluid force) as well as at the *t*_R_. *n* = 30 cells, 3 experiments, **p < 0.01 downstream edge at t = t_R_ relative to downstream edge at t = −10 min. (D) The percentage of VC-, BAPTA-AM-, EIPA-, BAPTA-AM+EIPA-, and ionomycin-treated HT-1080 cells that reverse their direction in tight confinement under flow conditions (4.8-nN fluid force); ≥30 cells per experiment, 3 experiments; **p < 0.01 relative to the VC, #*p* < 0.05, and $ $*p* < 0.01. (E) Image sequence of a tightly confined HT-1080 cell expressing optoGEF-RhoA and CAAX-CIBN-GFP after initiation of flow (4.8-nN fluid force). The yellow box indicates the region stimulated with blue light, and the green arrow points to the optoGEF-RhoA enrichment at the plasma membrane. Dotted lines show the cell position before and 20 min after its stimulation with blue light. Scale bar, 30 μm. (F) Migration velocity of HT-1080 cells expressing optoGEF-RhoA and CAAX-CIBN-GFP or only optoGEF-RhoA following exposure to a 4.8-nN fluid force and after their upstream or downstream stimulation with blue light. *n* ≥ 16 cells pooled from ≥16 experiments, *p < 0.05. (G) The percentage of HT-1080 cells expressing optoGEF-RhoA and CAAX-CIBN-GFP or only optoGEF-RhoA that reverse direction following exposure to a 4.8-nN fluid force and after their upstream or downstream stimulation with blue light. *n* ≥ 16 cells pooled from ≥16 experiments. (H) Normalized fluorescence intensity of pHrodo Red AM in tightly confined VC-, EIPA-, or LatA-treated HT-1080 cells under static conditions or following exposure to a 4.8-nN fluid force. The black arrow indicates the flow initiation time point. Values are normalized to the intensity of the same cell 15 min before flow initiation. *n* = 30 cells, 3 experiments; #*p* < 0.05 flow + LatA relative to flow + VC, $ $*p* < 0.01 static + VC relative to the flow + VC, **p < 0.01 flow + VC relative to static + VC, flow + EIPA, and flow + LatA. (I) Normalized upstream area of reversing VC- or EIPA-treated HT-1080 cells in tightly confined microchannels 15 min after flow initiation (4.8-nN fluid force). *n* ≥ 25 cells, 3 experiments; *p < 0.05. Statistical tests: unpaired Student’s t test (A, F, and I), one-way ANOVA (D), and two-way ANOVA (C and H) followed by Tukey’s multiple-comparisons post hoc test. Values represent mean ± SD (A, C, F, H, and I) or mean ± SEM (D). See also Figure S4.

Calcium serves as a secondary messenger, mediating cell responses to shear^40^ and pressure forces^21^ and activating actomyosin contractility.^21,40,41^ To monitor its intracellular levels, we employed the calcium indicator dye Fluo-4 Direct, whose intensity increases when intracellular calcium levels rise, as shown using the ionophore ionomycin (5 μM) (Figure S4A and S4B). Fluo-4 Direct fluorescence also rose rapidly in tightly confined cells subjected to a 4.8-nN fluid force (Figure 4A). These findings were corroborated using an alternative calcium reporter, the genetically encoded calcium indicator GCaMP6s (Figure 4A). The subcellular calcium distribution resembled that of myosin IIA; calcium levels increased downstream relative to upstream (Figures 4B and 4C). This differential increase was more pronounced at the *t*_R_ (Figure 4C). Chelating intracellular calcium with 1,2-bis(2-aminophenoxy)ethane N,N,N′,N′-tetraacetic acid acetoxymethyl ester (BAPTA-AM; 25 μM) reduced the number of upstream moving cells, while ionomycin treatment increased the extent of cell reversal (Figures 4D and S4C).

The downstream accumulation of both calcium and myosin II suggests the involvement of contractile forces in cell reversal. To determine whether these forces influenced upstream migration, we employed optogenetic tools that allowed us to tune contractility with high spatiotemporal accuracy.^42^ Blue light stimulation enables the binding of optoGEF-RhoA, a fusion protein consisting of the catalytic domain of the RhoA guanine nucleotide-exchange factors (GEF) activator ARHGEF11 and Cry2-mCherry, to CAAX-CIBN-GFP located on the plasma membrane.^42^ This binding triggers the local activation of RhoA, leading to an increase in contractile forces.^42^ Using HT-1080 cells expressing optoGEF-RhoA and CAAX-CIBN-GFP, we showed that light stimulation of the cell side exposed to lower pressure triggered the rapid accumulation of optoGEF-RhoA in that specific region (Figure 4E), leading to an enhancement in the number of cells moving upstream (Figures 4E–4G). In comparison, upstream activation of contractility nearly abolished cell reversal (Figures 4F and 4G). Exposing the upstream or downstream side of cells expressing optoGEF-RhoA but lacking CAAX-CIBN-GFP to blue light did not recruit optoGEF-RhoA to the plasma membrane (Figure S4D), thereby failing to alter the extent of cell reversal (Figures 4F and 4G).

Actin polymerization at the cell front enhances Na^+^/H^+^ exchanger 1 (NHE1) polarization via its actin-binding partner ezrin.^1^ NHE1 extrudes protons and imports sodium,^43^ thereby increasing intracellular pH and promoting a cell volume increase, which facilitates confined cell migration.^1,44^ The upstream polymerization of actin (Figures 3C and 3D) prompted us to investigate the role of NHE1 in fluid force-induced cell reversal. Using the pHrodo Red AM indicator dye, which becomes fluorescent when the intracellular pH decreased, we found that a 4.8-nN fluid force elevated the intracellular pH in tight confinement (Figures 4H and S4E), indicating enhanced NHE activity. This increase in intracellular pH was abolished by inhibiting NHE1 (via 5-(N-ethyl-N-isopropyl) amiloride (EIPA); 20 μM), or actin polymerization (Figure 4H). Moreover, EIPA decreased the upstream cell swelling (Figure 4I) and reduced the number of cells undergoing upstream migration (Figures 4D, S4C and S4F). Concurrent inhibition of NHE1, and intracellular calcium led to an even more pronounced decrease in fluid force-induced cell reversal (Figures 4D and S4C), suggesting that the actin-dependent activation of NHE1 and calcium cooperate to promote upstream cell motility.

### Lower levels of lamin A/C trigger downstream migration by suppressing tumor cell mechanosensitivity

**Figure 5.**
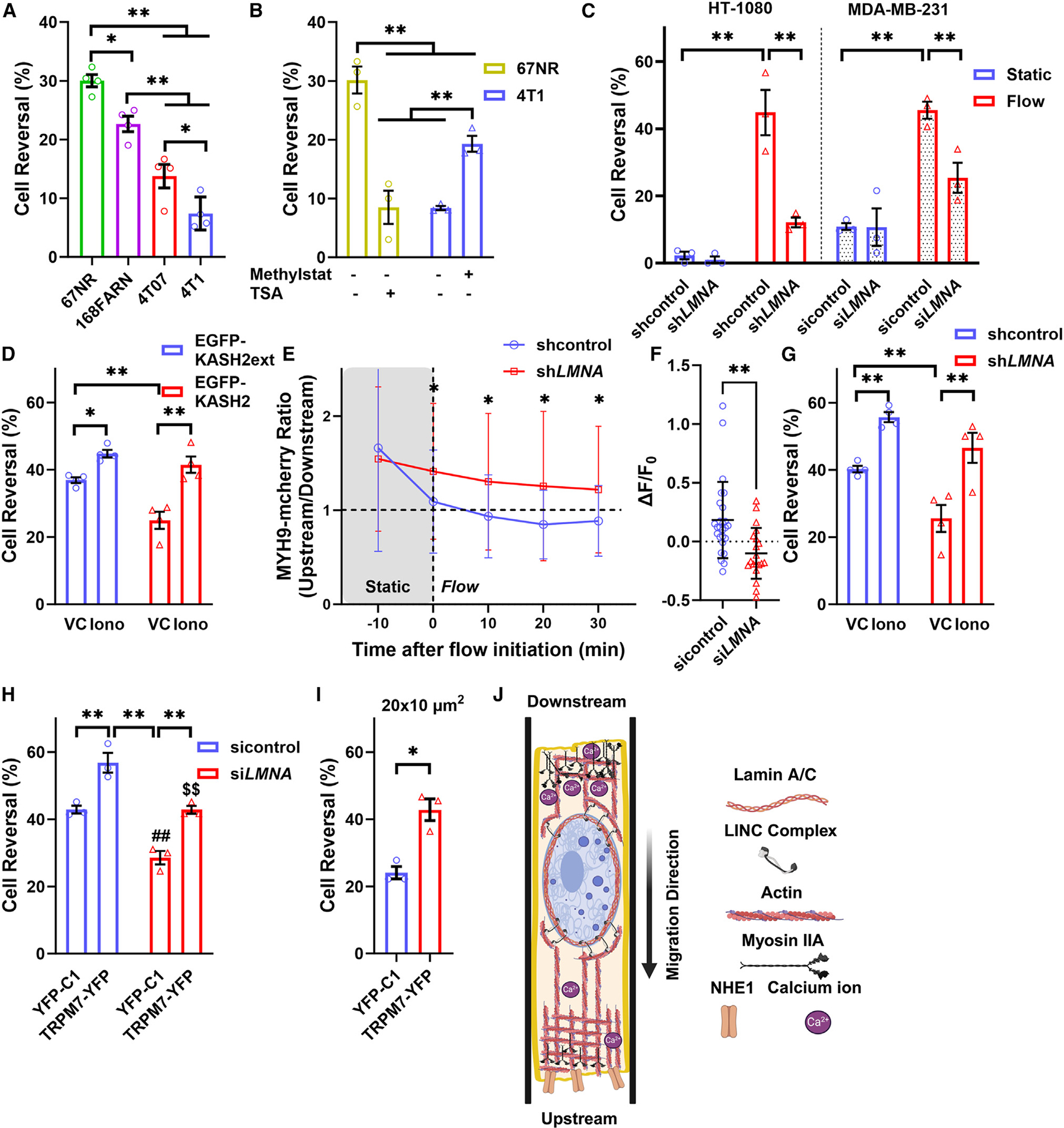
Reduced cancer cell mechanosensitivity mediates downstream migration (A–D) The percentage of cells that reverse their direction in tight confinement under flow conditions (4.8-nN fluid force). (A) 4T1, 4T07, 168FARN, and 67NR breast cancer cells; ≥28 cells per experiment, 4 experiments; *p < 0.05, **p < 0.01. (B) VCand TSA-treated 67NR breast cancer cells and VC- and methylstat-treated 4T1 breast cancer cells; ≥30 cells per experiment, 3 experiments; **p < 0.01. (C) shcontrol and sh*LMNA* HT-1080 cells and sicontrol and si*LMNA* MDA-MB-231 cells; ≥24 cells per experiment, 3 experiments, **p < 0.01. (D) HT-1080 cells expressing EGFP-KASH2ext or EGFP-KASH2 and treated with VC or ionomycin; ≥34 cells per experiment, 4 experiments; *p < 0.05, **p < 0.01. (E) Upstream-to-downstream ratio of MYH9-mCherry signal in shcontrol and sh*LMNA* HT-1080 cells before and after flow initiation (4.8-nN fluid force). *n* ≥ 29 cells, 3 experiments, *p < 0.05 sh*LMNA* relative to the shcontrol. (F) Quantification of Fluo-4 Direct fluorescence (ΔF/F_0_) in sicontrol and si*LMNA* HT-1080 cells in tightly confined microchannels following exposure to a 4.8-nN fluid force. *n* ≥ 20 cells, 3 experiments, **p < 0.01. (G and H) The percentage of (G) shcontrol and sh*LMNA* HT-1080 cells treated with VC or ionomycin and (H) sicontrol and si*LMNA* HT-1080 cells expressing TRPM7-YFP or YFP-C1 that reverse their direction following exposure to a 4.8-nN fluid force. (G) ≥16 cells per experiment, 4 experiments, **p < 0.01. (H) ≥24 cells per experiment, 3 experiments; ##*p* < 0.01 relative to sicontrol YFP-C1, $ $*p* < 0.01 relative to sicontrol TRPM7-YFP, **p < 0.01. (I) The percentage of HT-1080 cells expressing TRPM7-YFP or YFP-C1 that reverse their direction in moderate confinement under flow conditions (~5-nN fluid force). ≥10 cells per experiment, 3 experiments, *p < 0.05. (J) Schematic summarizing the mechanisms underlying upstream migration in tight confinement. Actin polymerizes upstream and is necessary for activation of NHE1, which promotes cell swelling and works together with calcium to induce migration toward higher pressure regions. Calcium accumulates downstream, mirroring the distribution pattern of myosin IIA, whose activation increases the proportion of cells moving upstream. A rigid nucleus, tethered to the cytoskeleton via the LINC complex, facilitates upstream migration by allowing cells to establish cell polarity and increase intracellular levels of calcium in response to fluid forces. Statistical tests: paired Student’s t test (I), unpaired Student’s t test (F), and one-way ANOVA (A) and two-way ANOVA (B, C, D, E, G, and H) followed by Tukey’s multiple-comparisons post hoc test. Values represent mean ± SD (E and F) or mean ± SEM (A–D and G–I). See also Figure S5.

For nearly three decades, it has been recognized that breast tumors display increased IFP relative to the adjacent healthy tissue.^27,45^ We thus hypothesized that highly metastatic breast tumor cells were more likely to escape high-pressure environments and move downstream than non-metastatic cells. To test this, we employed tumor cells from the isogenic 4T1 breast cancer progression series. In contrast to other breast cancer cell lines, which were highly heterogeneous (e.g., MDA-MB-231 cells^46,47^), cells from the 4T1 breast cancer progression series were derived from a single, spontaneously arising tumor in a BALB/cfC3H mouse and displayed distinct metastatic potential.^48,49^ The 168FARN, 4T1, and 4T07 cell lines were highly invasive, spreading to the lymph nodes (168FARN), lungs (4T07), or both lungs and bones (4T1), whereas the 67NR cells remained localized at the primary tumor. Our assay revealed that all invasive cell lines were more prone to move toward lower-pressure regions relative to their non-metastatic counterparts (Figure 5A). Notably, the highly metastatic 4T1 cells migrated almost exclusively downstream (Figure 5A).

Several highly invasive/metastatic breast cancer cell lines, including 4T1, 4T07, and 168FARN cells, express lower levels of A-type lamins compared to non-metastatic cells (e.g., 67NR).^50^ Because A-type lamins, such as lamin A/C, are key constituents of the nuclear envelope and promote nuclear rigidity,^51,52^ we investigated whether altering nuclear stiffness affected the directional responses of cancer cells to fluid forces. To this end, we treated highly metastatic 4T1 cells with methylstat (2 μM), a histone demethylase inhibitor that enhances nuclear rigidity through heterochromatin formation,^53^ and non-metastatic 67NR cells with trichostatin A (TSA; 100 ng/mL), a histone deacetylase inhibitor that decreases nuclear stiffness by increasing euchromatin.^54^ Methylstat-induced nuclear stiffening increased the number of upstream-moving 4T1 cells, whereas TSA-induced nuclear softening enhanced the fraction of 67NR cells migrating downstream (Figure 5B). Moreover, lamin A/C KD (si*LMNA* or sh*LMNA*) in HT-1080 or MDA-MB-231 cells (Figures S5A–S5C) promoted migration toward lower-pressure regions (Figures 5C, S5D, and S5E). Similar findings were also observed in HT-1080 cells expressing EGFP-KASH2 (Figure 5D), which acts as a dominant-negative nesprin, disrupting the linker of nucleoskeleton and cytoskeleton (LINC) complex and thereby the force transmission from the nucleus to the cell surface and vice versa.^55,56^ These data indicate that a rigid nucleus, tethered to the cytoskeleton via the LINC complex, is essential for sensing fluid forces and driving upstream migration in tight confinement.

Accumulated evidence suggests that lamin A knockout, or disconnecting the nucleus from the cytoskeleton, results in defects in cell polarity^55,57–59^ and loss of cellular tension^58,60,61^ that suppresses the activation of calcium-permeable mechanosensitive ion channels (MICs) and calcium influx.^1,21^ Consistent with this, fluid forces failed to trigger reversal of the myosin IIA polarity and elevation of intracellular calcium levels in tightly confined, lamin A/C KD cells (Figures 5E and 5F). Treatment with ionomycin increased the extent of cell reversal in lamin A/C KD or EGFP-KASH2-expressing cells to levels comparable to the control, indicating that calcium influx could restore their diminished mechanosensitivity (Figures 5D, 5G, and S5D). Similar results were obtained by overexpressing the calcium-permeable MIC TRPM7, which mediates shear and pressure sensing^21,40,62^ (Figure 5H). This intervention also triggered upstream migration in moderate confinement, suggesting that enhanced mechanosensitivity could outcompete the effects of chemokine gradients formed by flow (Figure 5I). In sum, lower lamin A/C levels and, consequently, increased nuclear deformability facilitate downstream migration of highly metastatic tumor cells by reducing their ability to establish cell polarity, activate MICs, and, thus, respond to fluid forces.

## DISCUSSION

This study elucidates the impact of pressure-driven flow on migration direction in spatially restricting environments. We show that fluid forces induce a significant shift in cell migration direction in tight, but not moderate, confinement, triggering cell movement toward higher-pressure regions. Counterintuitively, this fluid force-induced directional change occurs more frequently as fluid forces increase. Fluid forces elicit their effects via mechanisms involving key components of the migration machinery, such as the cytoskeleton, nucleus, and ion channels (Figure 5J). Mathematical modeling and experiments reveal that actin polymerization to the new cell front is required for cell reversal in tightly confined spaces. Upstream accumulation of actin likely elevates plasma membrane tension to counterbalance the increased fluid forces.^63^ Moreover, actin polymerization is essential for the fluid force-induced activation of NHE1, an ion transporter that facilitates confined migration by mediating cell volume increase.^1,5,11^ Inhibition of NHE1 activity reduces upstream cell swelling and limits the extent of cell reversal. The myosin IIA subcellular distribution differs from that of actin; myosin IIA accumulates downstream, and its depletion suppresses, but does not abolish, cell reversal. Intracellular calcium levels, which rapidly increase in response to fluid forces, mirror the same distribution pattern as myosin IIA, and together with NHE1, they cooperatively promote cell reversal. Because the calcium/calmodulin/myosin light-chain kinase pathway activates actomyosin contractility,^41^ downstream accumulation of calcium and myosin II may lead to the local activation of contractile forces, which induces upstream motility, as shown using optogenetics. Downstream polarization of calcium may also cause the disassembly of focal adhesions,^64,65^ further facilitating cell reversal.

Moderately confined cells experience both shear and pressure forces due to the absence of a tight seal between the cells and the channel walls. Tightly confined cells occupy the entire channel cross-sectional area, eliminating fluid flow. Hence, these cells sense only pressure drag. These findings align with previous research, indicating that a reduction in the pore size within a 3D matrix increases the pressure-to-shear drag ratio.^66^ Furthermore, we show that moderately confined cells fail to move upstream regardless of the fluid force magnitude tested. Also, a subset of cells in tightly confined channels consistently fails to reverse direction toward regions of higher pressure. These findings point to the presence of mechanisms that oppose fluid force-induced upstream migration. We demonstrate three such mechanisms: (1) chemotaxis, (2) hydraulic resistance, and (3) cell sensitivity to mechanical cues.

Downstream migration in 3D hydrogels occurs through autologous chemotaxis, an autocrine signaling mechanism in which chemokines (e.g., CCL19) secreted by cells and sensed via chemokine receptors (e.g., CCR7) form chemical gradients due to fluid convection.^36^ Moderately confined cells treated with a CCR7-blocking antibody move against the flow, resembling the directional behavior of tightly confined cells under the same flow conditions. These findings suggest that the reduced fluid flow, resulting from increased confinement, attenuates the formation of pericellular chemokine gradients, enabling fluid forces to initiate upstream cell migration. This is further supported by data indicating that blocking CCR7 has no impact on cell reversal in tight confinement, while downstream accumulation of chemoattractants outcompetes the effects of fluid forces on tightly confined cells, directing a significant fraction of cells toward lower-pressure regions.

In less restrictive environments, cells easily reverse their direction by displacing water around them. In tightly confined channels, cell reversal becomes more challenging because cells must push the column of water ahead of them.^67^ This is supported by data showing that cell reversal triggered by the 4.8-nN fluid force is diminished under conditions that elevate hydraulic resistance, such as high fluid viscosity or increased microchannel length. These findings are consistent with prior research, indicating that directional choices are tied to the resistance confined cells encounter.^19–22^ Although we employed polydimethylsiloxane (PDMS)-based, water-impermeable microchannels to explore the hydraulic resistance effects on upstream migration, depending on the local geometry, permeability and fluid viscosity magnitude, 3D matrices may introduce resistance levels equivalent to or higher than those observed in our experimental setup.^68^

Our data suggest that lamin A/C KD or LINC complex disruption results in loss of fluid force sensitivity, which, in turn, predominantly promotes downstream migration in tight confinement. This diminished mechanosensitivity can likely be attributed to the downregulation of RhoA/actomyosin contractility,^58,60,61^ which leads to lower intracellular tension,^69,70^ preventing cells from adequately balancing the elevated fluid forces. Reduced cytoskeletal and plasma membrane tension can also decrease the activity of MICs and calcium influx,^1,21^ further suppressing myosin II contractility. Moreover, the nucleus, tethered to the cytoskeleton via the LINC complex, regulates cell polarity.^55,57–59^ Consistent with these findings, we show that lamin A/C KD abolishes fluid force-induced intracellular calcium increase and myosin II repolarization. Overexpression of TRPM7, which mediates fluid force sensing,^21,40,62^ or enhanced calcium influx restores the compromised fluid force sensitivity of lamin A/C-depleted cells, presumably by activating actomyosin contractility.^21,40,41^ TRPM7 overexpression also increases the fraction of moderately confined cells undergoing upstream migration, suggesting that enhanced mechanoperception may be sufficient to initiate migration against the flow in less restrictive environments. Upstream 2D migration has been observed in certain cell types, such as fish epidermal keratocytes^71^ and ameboid cells like T cells,^72^ marginal zone B cells,^73^ and hematopoietic stem cells.^74^ In addition to cell mechanosensitivity, integrin-ligand interactions can influence the efficiency of upstream migration on planar surfaces, as demonstrated using different types of immune cells.^75^

Multiple cancer types display elevated IFP due to the increased permeability of a tumor’s vessels and the near absence of functional lymphatic vessels.^28,76,77^ The IFP drops significantly at the tumor’s edge, creating a pressure gradient that drives outward convection.^27^ While most cancer cells reside within the primary tumor, a smaller subset possesses the ability to evade the tumor mass and launch metastatic colonization. Although all cells in this study, whether cancerous or not, contain subpopulations capable of moving in the upstream and downstream direction, the downstream-moving cell subpopulation is more enriched in tumor cells with enhanced metastatic potential, such as those expressing lower levels of lamin A/C. TRPM7 overexpression, intracellular calcium rise, and nuclear stiffening emerge as promising strategies to inhibit the downstream migration of lamin A/C^low^ cancer cells and, thus, their escape from high-pressure environments, such as the primary tumor.

In sum, our research demonstrates that fluid forces restrict confined migration toward lower-pressure regions. However, downstream accumulation of chemoattractants, increased hydraulic resistance, and diminished fluid force sensitivity oppose the effects of fluid forces on confined cells, facilitating migration with the flow, which could enhance cancer cell invasion and metastasis.

### Limitations of the study

Our microfluidic devices provide precise control over the degree of cell confinement and flow rates, enabling us to investigate the effects of fluid forces on confined cell migration. However, experiments were carried out using stiff PDMS-based microchannels and −ΔP ranging from 0–240 Pa. It remains unknown how microchannel stiffness and ΔP outside of this range influence rheotactic cell behavior. While our work implicates TRPM7 in regulating upstream migration, we cannot rule out that other MICs may also participate in fluid force sensing and fluid force-dependent directional responses. New optogenetic constructs are needed to fully decipher NHE1 and MIC/calcium contributions to upstream migration. Although our results using breast cancer cell lines with varying metastatic potential suggest a link between downstream migration and metastatic spread, further research is necessary to elucidate the role of fluid forces in cancer cell migration, invasion, and metastasis *in vivo*.

## RESOURCE AVAILABILITY

### Lead contact

Further information and requests for resources and reagents should be directed to and will be fulfilled by the lead contact, Panagiotis Mistriotis (pmistriotis@auburn.edu).

### Materials availability

This study did not generate new unique reagents.

### Data and code availability

- All data reported in this paper will be shared by the lead contact upon request.
- The code to model F-Actin, G-Actin, and Myosin has been deposited on GitHub (https://zenodo.org/doi/10.5281/zenodo.13145692).
- Any additional information required to reanalyze the data reported in this paper is available from the lead contact upon request.

## STAR★METHODS

### EXPERIMENTAL MODEL AND STUDY PARTICIPANT DETAILS

#### Cell lines

MDA-MB-231 breast cancer cells (NCI-PBCF-HTB26) as well as adipose- and bone marrow-derived mesenchymal stem cells were purchased from the American Type Culture Collection (ATCC). Wild type HT-1080 fibrosarcoma cells, HT-1080 cells expressing LifeAct-GFP and H2B-mChery, HT-1080 cells expressing optoGEF-RhoA and CAAX-CIBN-GFP, HT-1080 cells expressing EGFP-KASH2ext or EGFP-KASH2, HT-1080 cells expressing YFP or TRPM7-YFP, as well as scramble control, sh*MYH9* and sh*LMNA* HT-1080 cells were gifted by K. Konstantopoulos (Johns Hopkins University). Tumor cells from the isogenic 4T1 breast cancer progression series were gifted by Dr. Friedl (Radboud University Nijmegen Medical Center). All cell lines have been tested negative for mycoplasma with PCR (forward primer: GGGAGCAAACAGGATTAGATACCCT, reverse primer: TGCACCATCTGTCACTCTGTTAACCTC).

### METHOD DETAILS

#### Cell culture and pharmacological inhibitors or activators

HT-1080 and MBA-MB-231 cells were cultured in Dulbecco’s modified Eagle’s medium (DMEM; Gibco) that is supplemented with 10% (v/v) fetal bovine serum (FBS; Bio-Techne) and 1% (v/v) penicillin/streptomycin (10,000 U/mL; Gibco). 4T1 progression series cell lines were maintained in Roswell Park Memorial Institute medium (RPMI) 1640 supplemented with L-glutamine (Quality Biological), 10% (v/v) FBS (Bio-Techne) and 1% (v/v) penicillin/streptomycin (10,000 U/mL; Gibco). Mesenchymal stem cells were cultured in Mesenchymal Stem Cell Basal Medium for Adipose, Umbilical and Bone Marrow-derived MSCs (ATCC) that is supplemented with either Mesenchymal Stem Cell Growth Kit for Bone Marrow-derived MSCs or Mesenchymal Stem Cell Growth Kit for Adipose and Umbilical-derived MSCs (ATCC). All cell lines were cultured in an incubator at 37°C and 5% CO_2_ and passaged every 2 to 5 days. Select experiments have been performed using the following pharmacological agents and appropriate vehicle controls: Blebbistatin (BioVision; 20 μM), Y27632 (Sigma-Aldrich; 10 μM), Latrunculin A (R&D Systems; 2 μM), Ionomycin (Sigma-Aldrich; 5 μM), BAPTA-AM (Sigma-Aldrich; 25 μM), EIPA (Enzo Life Sciences; 20 μM), Trichostatin A (Sigma-Aldrich; 100 ng/μL), and Methylstat (Sigma-Aldrich; 2 μM).

#### Photolithography and microchannel device fabrication

Soft photolithography and replica molding were used to fabricate PDMS-based microchannel devices of different dimensions.^39,61^ First, a layer of SU-8 3010 negative photoresist (Microchem) with a thickness of 3 or 10 μm was deposited on a prime grade 4″ silicon wafer (University Wafer). The wafer was then exposed to UV light through a chrome-on-glass darkfield photolithography mask featuring 10- or 20-μm wide microchannels. After removing the uncrosslinked SU-8 3010, a second layer of SU-8 3025 with a thickness of 50 μm was deposited on top of the microchannels and exposed to UV light through a mask featuring the larger, 2D-like channels. The width and height of channels were verified using a profilometer. The completed wafers were used as molds to prepare microchannel devices. PDMS prepolymer and curing agent were mixed at a 10:1 ratio, poured over the wafer and cured at 85°C for 80 min. Polymerized PDMS was peeled from the wafer mold, plasma cleaned and bound to a plasma cleaned 75-mm glass coverslip. All microfluidic devices were coated with rat tail collagen type I (Corning; 20 μg/mL) for 1 h at 37°C.

#### Calculation of ΔP

For moderately confined (*W(idth) × H(eight)* = 20 × 10 μm^2^) and tightly confined (*W × H* = 10 × 3 μm^2^) microfluidic devices the pressure differential needed to generate a certain fluid velocity within the microchannels is calculated by^82^:

(Equation 1) $$\Delta P_{channel}=\frac{12\mu Lq}{WH^{3}}{\left[1-\frac{192H}{\pi^{5}W}\right]}^{-1}$$

Where $\Delta P_{channel}$ is the pressure drop across a single channel, μ is the viscosity of the fluid inside the channel, q is the volume flow rate through a single channel, and L,W *and* H are the length, the width and height of the microchannel.

From Equation 1, we can calculate the resistance of a single microfluidic channel as:

(Equation 2) $$R=\frac{12\mu L}{WH^{3}}{\left[1-\frac{192H}{\pi^{5}W}\right]}^{-1}$$

To calculate the equivalent resistance of the entire microfluidic device, we considered the resistances of the lowermost and uppermost channels as well as the effective resistance of the microchannel array as shown in Figure S6A. The resistances of the uppermost and lowermost channels are calculated by Equation 2. The effective resistance of the microchannel array is $R_{\text{Microchannels}}=R/N$, where N is the number of microchannels in the array. The equivalent resistance of the entire device is calculated by:

(Equation 3) $$R_{\text{eq}}=R_{\text{Lowermost}}+R_{\text{Uppermost}}+R_{\text{Microchannels}}$$

The total volume flow rate across the device is:

(Equation 4) $$Q=\frac{\Delta P}{R_{eq}}$$

where ΔP is the pressure differential across the device. The average flow velocity in a single channel is thus calculated by:

(Equation 5) $$\overset{‾}{v}=\frac{Q}{NWH}$$

#### Imaging and velocity analysis of microbeads

To quantify fluid velocity, 1 μm diameter Fluoro-Max Dyed Green Aqueous Fluorescent Particles (Thermo Scientific- Microgenics Corporation) were used. These microbeads were introduced into the cell culture medium at a concentration of 0.15% v/v in both the inlet and outlet wells of the device. They were then allowed to flow under the influence of prescribed ΔP. Time-lapse imaging of flowing microbeads was conducted using a Prime BSI Express high-speed camera mounted on a Nikon Eclipse Ti2 Inverted microscope (Nikon) at a frame rate of 5–40 frames per seconds. To accurately represent the fluid velocity profile within moderately and tightly confined microchannels, a selection of 40–60 beads was made, ensuring that the entire width and height of the microchannels were covered. The velocity of beads within the taller, moderately confined channels was also validated using an A1R resonance scanning microscope. Considering the bead size (1 μm), the estimated diffusion coefficient for the bead falls in the range of 0.1 μm^2^/s. The effective velocity associated with the diffusion is on the order of 1 nm/s, a value that can be deemed negligible in comparison to the flow velocity. Microbead tracking was carried out using the ImageJ plugin MTrackJ, and microbead velocity was calculated using a custom-made MATLAB script (MathWorks, Natick, MA).

#### Cell seeding and generation of a pressure gradient

Cells were resuspended in DMEM supplemented with 1% (v/v) penicillin/streptomycin at a concentration ranging from 5 × 10^5^–5 × 10^6^ cells/mL. 20 μL of this cell suspension was used to seed cells near the entrances of the microchannels. The devices were then incubated at 37°C for 10–15 min to facilitate cell adhesion to the collagen type I-coated surface.

In experiments assessing cell reversal, initially, cells were allowed to migrate through moderately or tightly confined microchannels in an isobaric environment toward a chemoattractant (10% v/v FBS) placed in the uppermost 2D-like channel. After approximately 2 h, the existing medium within the device was replaced with fresh cell culture medium containing 10% FBS. Fluid flow was initiated by manipulating the height of the medium in the inlet wells relative to the outlet wells, thereby creating a ΔP between the lowermost and uppermost 2D-like channel. This ΔP was determined using the expression ΔP=ρgΔh, as shown in the Table below, where r represents the density of water (1,000 kg/m^3^), g is the gravitational acceleration (9.81 m/s^2^) and Δh represents the vertical variation in height between the medium levels at the inlet and outlet wells. Note that at the time of flow initiation, cells were randomly distributed within various segments of the microchannels.

**Table:** 

**The height difference and the corresponding pressure differentials**											

~Δh (cm)	0.02	0.06	0.10	0.12	0.24	0.37	0.41	0.49	0.82	1.63	2.45
ΔP(Pa)	1.5	6	10	12	24	36	40	48	80	160	240

In experiments assessing the role of CCR7 in fluid force-induced cell reversal, cells were first allowed to adhere to the collagen type I-coated surface of moderately and tightly confined devices for 1 h. Subsequently, cells were incubated at 37°C for 2 h in media containing either the CCR7 blocking antibody (5 μg/mL; R&D Systems) or the Mouse IgG2A Isotype Control (5 μg/mL; R&D Systems). Flow was then initiated using media containing either the blocking antibody or the isotype Control.

In experiments evaluating cell entry into microchannels, pressure-driven flow was initiated immediately following cell adhesion to the surface. Cell entry experiments were carried out in the absence of any chemoattractant. Directional responses to fluid forces were recorded via Flash 4.0 camera mounted on a Nikon Eclipse Ti2 Inverted microscope (Nikon). Throughout the migration experiments, cells were maintained at a controlled environment of 37°C with 5% CO_2_ and 100% humidity, achieved through the use of a stage top incubator (Tokai Hit Co.).

The durations of cell entry and cell reversal experiments were determined after evaluating how the microbead velocity changed with time. Because fluid velocities stayed within 15% of initial levels during the initial 3-h period after the onset of flow (Figure S6B), experiments aimed to compare cell reversal in distinct microchannel sizes were conducted during this time frame. Experiments assessing cell entry into tightly confined microchannels were carried out for 6 h.

#### Quantification of cell reversal, cell entry, and migration velocity

The percentage of cell reversal in response to fluid forces was determined by dividing the number of cells that reversed their directions with the total number of cells in the microchannels. Cells interacting with other cells in the microchannels, dividing cells, cells touching the michrochannel’s end, cells with no movement, and apoptotic cells were excluded from the analysis. A cell is reversing if its overall displacement within the microchannel is negative and non-reversing if it’s positive. The percentage of cell entry was determined by dividing the number of cells that entered tightly confined microchannels with the total number of cells located within 50 μm of the channel entrance. Cell migration tracking was conducted using MTrackJ and cell velocity was calculated using a custom-made MATLAB script.

#### siRNAs and plasmid transfection

For transient knockdown experiments, HT-1080 and MDA-MB-231 cells were transfected with siRNAs targeting scramble (sc-37007; Santa Cruz Biotechnology) and lamin A/C (sc-35776; Santa Cruz Biotechnology). Transfections were carried out using Lipofectamine RNAiMAX Transfection Reagent (13778150, Invitrogen) following the prescribed manufacturer’s protocol. Myosin-IIA-GFP^79^ was a gift from Matthew Krummel (Addgene plasmid #38297), pCMV-mCherry-MHC-IIA^80^ was a gift from Venkaiah Betapudi (Addgene plasmid #35687), and pGP-CMV-GCaMP6s^81^ was a gift from Douglas Kim & GENIE Project (Addgene plasmid #40753).

#### Optogenetic activation of contractile forces

HT-1080 cells expressing CAAX-CIBN-GFP and optoGEF-RhoA were allowed to migrate through tightly confined microchannels under static conditions. The microfluidic devices were then placed on a Nikon AXR confocal microscope equipped with a stage top incubator and fluid flow was initiated as described above. Cell migration was then recorded for 5 min using the mCherry channel. A 1% blue light laser (488 nm) was used to stimulate a small rectangular region of interest (ROI) near the cell edge facing high- or low-pressure. Stimulations were applied 20 times, each lasting 1 s, with a 10-s interval between each stimulation. Subsequently, stimulated cells were monitored for a period of 20 min using the mCherry channel to track the localization of ARHGEF11 and determine the direction of cell migration. Control experiments were carried out using HT-1080 cells expressing only optoGEF-RhoA.

#### Analysis of cell morphology and phenotype

The ImageJ software’s polygon selection tool was utilized to create a closed ROI around the cell periphery. Next, the measure ROI tool was used to calculate the cell area, perimeter, and aspect ratio. The recorded values of each metric were normalized by dividing them with the corresponding value of the same cell recorded at the time of flow initiation. To quantify the upstream and downstream areas, we measured the area between the nucleus and the cell edge facing the upstream and downstream direction in ImageJ. For phenotypic quantifications, we assigned a value to both the leading and trailing edges of cells. A value of 0.5 was assigned for blebbing edges, while mesenchymal edges were assigned a value of 0. If both edges exhibited blebbing, the cell received a phenotypic score of 1; conversely, if both showed mesenchymal characteristics, the assigned score was 0. If only one edge displayed blebbing, the score was set at 0.5.

#### Fluorescence imaging and image quantification

HT-1080 cells expressing MYH9-GFP, mCherry-MYH9, or LifeAct-GFP/H2B-mCherry were allowed to migrate through moderately or tightly confined microchannels under static conditions. Approximately 5 min prior to the onset of flow, GFP or TRITC images were recorded using a Nikon Eclipse T*i*2 Inverted microscope to assess the localization of actin or myosin IIA in cells. Fluid flow was then initiated, and fluorescence imaging was conducted over a 3-h period, with intervals of either 10 or 15 min.

At each time point, the background-subtracted, upstream and downstream mean intensities of MYH9-GFP, LifeAct-GFP, or mCherry-MYH9 were quantified in ImageJ by drawing ROIs of fixed areas that encompass the cell front and rear. These values were then used to calculate the upstream to downstream ratio of the fluorescence signal.

#### Quantification of intracellular pH changes

pHrodo Red AM (Invitrogen) was utilized to monitor intracellular pH changes in live cells following the manufacturer’s instructions. Cells that had already been introduced into the microchannels were treated with pHrodo Red AM for 30 min at 37°C and 5% CO_2_. Subsequently, the dye solution was washed away, and Live Cell Imaging Solution (Gibco) was added. The cells were then kept under static conditions for 15 min. Next, the devices were placed on a Nikon AXR confocal microscope equipped with a Tokai Hit stage-top incubator. Images were captured once under static conditions and then every 15 min for 45 min under flow conditions using a 60X oil objective and a 0.2% 488-nm laser power. ImageJ software was employed to measure the background-subtracted mean fluorescence intensity of pHrodo Red AM by outlining regions of interest (ROIs) around the cell periphery.

#### Preparation of media with elevated viscosity and viscosity measurements

The viscosity of the cell culture media was increased by adding the prescribed amount of Methylcellulose stock solution (3%, R&D Systems) to Iscove Modified Dulbecco Media (IMDM; Gibco) supplemented with 10% (v/v) FBS and 1% (v/v) penicillin/streptomycin (10,000 U/mL), resulting in a final solution concentration of 0.6%.

Cell culture media viscosity was obtained using an Anton Paar MCR301 rotational rheometer at 37°C. A coaxial cylinder geometry with a 26.65 mm bob diameter, 28.92 mm cup inner diameter, and 39.98 mm gap length was utilized. Flow curve was generated in the shear rate ranging from 1 s^−1^ to 100 s^−1^ and two loads were performed. The time required to achieve steady state for the sample was determined via a transient test; the sampling time required to get the flow curve was reduced logarithmically as the shear rate increased. The fluid was Newtonian with an average viscosity of 11.2 cP.

#### Western blot

HT-1080 and MDA-MB-231 cells were lysed using the RIPA cell lysis buffer (Thermo Scientific) to extract the total protein. Protein quantification was carried out using the Bradford assay (Bio-Rad Laboratories). Proteins were reduced with 2x Laemmli Sample Buffer (Bio-Rad Laboratories) and equal amounts of protein (30 μg) were separated by SDS-PAGE using 10% Mini-PROTEAN TGX Precast Gels (Bio-Rad Laboratories). The Fisher brand Semidry Blotting Apparatus was used to transfer the proteins onto a PVDF membrane, which was then incubated in a blocking buffer (tris-buffered saline with 1% Casein) to prevent non-specific antibody binding. The membrane was subjected to overnight incubation at 4°C with primary antibodies specific to the protein of interest. This was followed by a 2-h incubation at room temperature with the corresponding secondary antibody. Amersham ECL Select Western Blotting Detection Reagent (Cytiva life sciences) and a ChemiDoc Imaging Systems (Bio-Rad Laboratories) were used to visualize the protein bands. The following antibodies were used: β-Actin (mouse; Sigma-Aldrich; 1:10,000; A3854), anti–lamin-A/C (mouse; 1:2000; Cell Signaling; 4777), anti-Myosin IIA antibody (rabbit; 1:1000; Sigma Aldrich; M8064). Secondary antibodies: anti-mouse immunoglobulin G (IgG), horseradish peroxidase (HRP)–linked antibody (goat; 1:10000; Cell Signaling; 7076S), and anti-rabbit IgG, HRP-linked antibody (goat; 1:10000; Cell Signaling; 7074S).

#### Calcium imaging and quantification

To quantify intracellular levels of calcium, we employed two well-established calcium indicators, namely the Fluo-4 Direct Calcium Assay Kit (Invitrogen) and the genetically encoded calcium indicator GCaMP6s. Cells already adhered to the surface of our microfluidic devices were treated with the Fluo-4 Direct dye for 45 min at 37°C and 5% CO_2_ followed by a 15-min incubation at room temperature. The dye was then replaced with regular cell culture media and cells were allowed to migrate through microchannels under static conditions. Next, the devices were placed on a Nikon AXR confocal microscope equipped with a Tokai Hit stage-top incubator and imaged every 6 s for 1 min under static conditions and every 6 s for 3 min under flow conditions using a 40X objective and a 20% 488-nm laser power. ImageJ software was used to measure the background-subtracted mean fluorescence intensity of Fluo-4 Direct by drawing ROIs around the cell periphery.

Tightly confined HT-1080 cells expressing GCaMP6s were imaged every 1 min for 90 min under static and flow conditions using the Nikon AXR confocal microscope. The background-subtracted mean fluorescence intensity of the whole cell as well as the upstream and downstream cell edges were measured using ImageJ. This analysis was conducted at different time points: before and immediately after flow initiation as well as at the cell reversal time point.

#### Estimation of fluid forces on tightly confined and moderately confined cells

We estimate the total fluid forces on cells in microfluidic channels of length L, height H, and width W. There are two types of fluid forces. The first is the force due to the pressure drop across the cell length, $\Delta P_{\text{cell}}$. The cross-sectional area of the cells perpendicular to the channel axis, $WH_{\text{cell}}$, is the area of projection on which the pressure applies (Figure S6C), where $H_{\text{cell}}$ is the effective height of the cell. Therefore, $F_{\text{pressure}}=WH_{\text{cell}}\Delta P_{\text{cell}}$. The second is the force due to the fluid viscous shear stress on the cells, $\tau_{\text{cell}}$. The projected 2D dorsal area of the cell, $A_{\text{cell}}=WL_{\text{cell}}$, is the area on which the shear stress applies, where $L_{\text{cell}}$ is the effective length of the cell. Therefore, $F_{\text{shear}}=A_{\text{cell}}\tau_{\text{cell}}$. The total fluid force on the cell is then $F_{\text{total}}=F_{\text{pressure}}+F_{\text{shear}}$.

In the experiments, pressure drops, ΔP, are applied across the microfluidic devices. Since the hydraulic resistance of the microchannels dominates the resistance of uppermost and bottommost 2D-like channels, we use ΔP to approximate the pressure drop across each channel, $\Delta P_{\text{channel}}$, of length L.

##### (1) Tightly confined cells

Cells in W=3μm by H=10μm channels are confined and occupy the entire cross-sectional area of the channels so that the cell height, $H_{\text{cell}}$, is identical to the channel height. Then the height of the gap space above the cell (Figure S6C) is zero, i.e., $H_{\text{gap}}=H-H_{\text{cell}}=0$. In this case, the cells do not experience shear stress, and only experience the force from the pressure drop across the channel. Therefore, the total fluid force on the cells is:

(Equation 6) $$F_{\text{total}}=F_{\text{pressure}}=HW\Delta P_{\text{channel}}$$

For example, if $\Delta P_{\text{channel}}=160\;Pa$, then $F_{\text{total}}=4.8\;n$.

##### (2) Moderately confined cells

Cells in W=20μm by H=10μm channels are moderately confined. A side view of a cell within a channel is illustrated in Figure S6C. Cells can display different morphologies when sitting in moderately confined spaces. For the purpose of calculation, we approximate the cell spreading area as a rectangle with width identical to the channel width, W. We can then approximate the effective cell length through measured cell area, i.e., $L_{\text{cell}}=A_{\text{cell}}/W$. The effective cell height is unknown, and we will solve it.

The cell separates the rectangular channel into two regions of different fluid hydraulic resistances. One region is the rectangular gap space above the cell. This space has a length of $L_{\text{cell}}$, a width of W, and a height of $H_{\text{gap}}$. The second region is the free space without a cell. This space has a length of $\left(L-L_{\text{cell}}\right)$, a width of W, and a height of H. The conservation of mass requires that the volume flow rate, q, in the two regions are the same. The volume flow rate can be calculated from the measured average bead velocity $V_{\text{bead}}$, i.e., $q=HWV_{\text{bead}}$.

For any rectangular channel of height H and width W, the volume flow rate relates to the pressure drop across the channel through $\Delta P_{\text{channel}}=qR$, where R is the hydraulic resistance of the channel and is calculated from Equation 2. We can use this equation to calculate the hydraulic resistance of the two regions, $R_{\text{gap}}$ and $R_{\text{free}}$, for any value of the effective cell height. The total hydraulic resistance of a channel with a cell is thus $R_{\text{total}}=R_{\text{gap}}+R_{\text{free}}$, which is a function of the unknown effective cell height, $H_{\text{cell}}$. The effective height of the cell is numerically solved by:

(Equation 7) $$f\left(H_{\text{cell}}\right)=qR_{\text{total}}\left(H_{\text{cell}}\right)-\Delta P_{\text{channel}}=0$$

Once $H_{\text{cell}}$ is obtained, the pressure drop across the cell is then

(Equation 8) $$\Delta P_{\text{cell}}=qR_{\text{gap}}$$

The viscous shear stress on the cell is calculated by^83^:

(Equation 9) $$\tau_{\text{cell}}=\frac{2\mu q}{WH_{\text{gap}}^{2}}\left(\frac{m+1}{m}\right)(n+1)$$

where $m=1.7+0.5\alpha^{-1.4}$ and n=2 if α≤1/3, and n=2+0.3(α-1/3) if α>1/3^84^, where $\alpha =H_{\text{gap}}/W$ is the aspect ratio. With $\Delta P_{\text{cell}}$ and $\tau_{\text{cell}}$ obtained, we then know the force on the cell due to the pressure drop and the shear stress, respectively.

#### A dynamic model for confined cell migration

The model considers cells migration in a one-dimensional confined channel of length L, width w, and height h (Figure S6D). The cells are confined horizontally so that h<w. The domain of the channel is x∈[0,L]. The front and back position of the cell are described by $x^{f}(t)$ and $x^{b}(t)$, respectively. Throughout the model, we use the scripts ‘f’ and ‘b’ to denote quantities associated with the front and back of the cell, respectively. $L_{c}(t)=x^{f}(t)-x^{b}(t)$ is the length of the cell (Figure S6D). The cell separates the channel into a front part and a back part, with respective lengths of $L_{f}(t)$ and $L_{b}(t)$.

The model includes fluid (cytosol and the extracellular fluid), actin, myosin, and solutes. The symbols used in the model are as follows: cytosol pressure, velocity, and viscosity: $p_{c}(x,t)$, $v_{c}(t)$, and $\mu_{c}$. Extracellular fluid pressure and viscosity in the front part of the channel: $p_{f}(x,t)$ and $\mu_{f}$. The counterparts at the back part of the channel are defined accordingly with the subscript ‘f’ replaced by ‘b’. F-actin velocity and concentration: $v_{n}(x,t)$ and $\theta_{n}(x,t)$. G-actin concentration and diffusion coefficient in the cytosol: $\theta_{c}(x,t)$ and $D_{\theta_{c}}$. Concentration of activated and non-activated myosin in the cell: $m_{n}(x,t)$ and $m_{c}(x,t)$. The respective diffusion coefficients are $D_{m_{n}}$ and $D_{m_{c}}$. Solute concentration in the cytosol: $c_{c}(x,t)$. Solute concentrations in the extracellular fluid in the front and back part of the channel: $c_{f}(x,t)$ and $c_{b}(x,t)$. The diffusion coefficients of the solute in the cytosol and the extracellular fluid are $D_{c_{c}},\;D_{c_{1}}$, and $D_{c_{b}}$.

##### Fluid module

At low Reynolds number, we can neglect the inertia term of the fluid. Therefore, the conservations of momentum and mass for the one-dimensional cytosol flow are

(Equation 10) $$0=-\frac{\partial p_{c}}{\partial x}-\frac{12\mu_{c}}{\tilde{a}h^{2}}v_{c}-\eta_{it}\theta_{n}\left(v_{c}-v_{n}\right).\;0=\frac{\partial v_{c}}{\partial x}.$$

where $\eta_{it}$ is the interfacial frictional factor due to the relative velocity between the cytosol and F-actin,^85^ and $\tilde{a}=1-192h/\left(\pi^{5}w\right)$ is a geometrical correction factor when h<w but h≪w is not satisfied.^82^ The continuity condition indicates that the cytosol velocity is a constant in space.

The pressure gradient in the back and front channel can be solved by a pipe flow,^68^

(Equation 11) $$\begin{array}{c} {\left.p_{b}\right|}_{x=x^{b}}={\left.p_{b}\right|}_{x=0}-\frac{12\mu_{b}L_{b}}{\tilde{a}h^{2}}v_{c},{\left.p_{b}\right|}_{x=0}=p_{b,}^{0}\\ {\left.p_{f}\right|}_{x=x^{f}}={\left.p_{f}\right|}_{x=L}+\frac{12\mu_{f}L_{f}}{\tilde{a}h^{2}}v_{c},{\left.p_{f}\right|}_{x=L}=p_{f}^{0}, \end{array}$$

where $p_{b}^{0}$ and $p_{f}^{0}$ are the extracellular fluid pressure at x=0 and x=L, respectively. The continuity condition requires that the fluid velocities inside and outside of the cell are the same.

The plasma membrane is permeable to water. The water influxes at the front and back ends of the cell are

(Equation 12) $$\begin{array}{c} J_{\text{water}}^{f}=-\alpha_{f}\left[\left({\left.p_{c}\right|}_{x=x^{f}}-{\left.p_{f}\right|}_{x=x^{f}}\right)-RT\left({\left.c_{c}\right|}_{x=x^{f}}-{\left.c_{f}\right|}_{x=x^{f}}\right)\right],\\ J_{\text{water}}^{b}=-\alpha_{b}\left[\left({\left.p_{c}\right|}_{x=x^{b}}-{\left.p_{b}\right|}_{x=x^{b}}\right)-RT\left({\left.c_{c}\right|}_{x=x^{b}}-{\left.c_{b}\right|}_{x=x^{b}}\right)\right], \end{array}$$

where $\alpha_{f}$ and $\alpha_{b}$ are the hydraulic conductance of the plasma membrane at the front and back of the cell, respectively. *RT* is the product of the ideal gas constant and the absolute temperature.

The membrane velocities at the two ends of the cell are related to the water flux and fluid velocity by

(Equation 13) $$\frac{dx^{f}}{dt}=v_{c}+J_{\text{water}}^{f}\frac{dx^{b}}{dt}=v_{c}-J_{\text{water}}^{b},$$

As the membrane moves, membrane tension is developed along the membrane. This tension is mostly attributed to the cell cortex beneath the membrane.^67^ We combine the plasma membrane and the cell cortex into a membrane-cortex complex. This membrane-cortex complex can be modeled as a visco-elastic material. Therefore, the membrane-cortex tension at the front and back ends of the cells are approximated by

(Equation 14) $$\tau^{f}=\eta_{m}\frac{dx^{f}}{dt}+k_{ct}\left(\frac{L_{c}\;-\;L_{c,0}}{L_{c,0}}\right),\tau^{b}=-\eta_{m}\frac{dx^{b}}{dt}+k_{ct}\left(\frac{L_{c}\;-\;L_{c,0}}{L_{c,0}}\right),$$

where $\eta_{m}$ and $k_{ct}$ are the membrane-cortex complex viscosity and stiffness, respectively, and $L_{c,0}$ is a reference cell length.

The actin network, formed by F-actin and activated myosin, exhibits fluid-like behavior. The presence of actin filaments creates a passive swelling stress, $\sigma_{n}$, which can be modeled by a linear constitutive relation, $\sigma_{n}=k_{\sigma_{n}}\theta_{n}$, where $k_{\sigma_{n}}$ is the coefficient of actin swelling. The activated myosin provides active contractile stress,^86,87^ $\sigma_{a}$, which depends on the concentration of activated myosin. Without loss of generality, we let $\sigma_{a}=k_{\sigma_{a}}m_{n}$, where $k_{\sigma_{a}}$ is the coefficient of myosin contraction. We can thus express the total stress in the actin network as $\sigma =\sigma_{n}-\sigma_{a}$^88^.

By neglecting the inertia of the membrane complex, the force balance at the front and back ends of the cell is given by

(Equation 15) $$\begin{array}{r} h\left[{\left.p_{f}\right|}_{x=x^{f}}-{\left.\left(\sigma +p_{c}\right)\right|}_{x=x^{f}}\right]=-2\tau^{f}+hf_{ext}^{f}-k_{ad}\frac{dx^{f}}{dt},\\ h\left[{\left.p_{b}\right|}_{x=x^{b}}-{\left.\left(\sigma +p_{c}\right)\right|}_{x=x^{b}}\right]=-2\tau^{b}+hf_{ext}^{b}+k_{ad}\frac{dx^{b}}{dt}, \end{array}$$

where $k_{\text{ad}}$ is the coefficient of adhesive force on the cell membrane due to substrate adhesive molecules. This adhesive force is physically similar to a frictional force on the cell. $f_{\text{ext}}$ are externally applied forces to the two ends of the cell. These two forces are positive in the outward normal direction.

##### Actin module

The conservation of momentum for the F-actin phase is

(Equation 16) $$-\frac{\partial \sigma }{\partial x}+\eta_{\text{it}\;}\theta_{n}\left(v_{c}-v_{n}\right)-\eta_{st}\theta_{n}v_{n}=0,$$

where $\eta_{st}$ is the strength of focal adhesion. The force from focal adhesion is modeled as an effective body force on the cell actin network.^68^ The conservation of mass for F-actin is

(Equation 17) $$\frac{\partial \theta_{n}}{\partial t}+\frac{\partial }{\partial x}\left(\theta_{n}v_{n}\right)=-\gamma \theta_{n},$$

where γ is the rate of actin depolymerization, which accounts for the interchange between F-actin and G-actin.^89^ In the absence of myosin, actin depolymerizes at a baseline rate, $\gamma_{0}$. In the presence of myosin, the active contraction from myosin within the actin network promotes actin depolymerization. Therefore, we model the rate of actin depolymerization as,^88^

(Equation 18) $$\gamma =\gamma_{0}+\gamma_{a}\sigma_{a}^{2},$$

where $\gamma_{a}$ is a myosin associated rate of actin depolymerization.

The boundary conditions for the actin network follows the conservation of mass at the boundaries:

(Equation 19) $${\left(\theta_{n}v_{n}\right)}_{x^{f}}=\theta_{n}^{f}\frac{dx^{f}}{dt}-J_{\text{actin}}^{f},{\left(\theta_{n}v_{n}\right)}_{x^{b}}=\theta_{n}^{b}\frac{dx^{b}}{dt}+J_{\text{actin}}^{b},$$

where $J_{\text{actin}}^{f}$ and $J_{\text{actin}}^{b}$ are the rate of actin polymerization at the front and back of the cell, respectively. In our prior models,^1,2,11,68,85,88,89^ actin polymerization was only applied to one end of the cell designated as the cell front. In this model, to consider a more general case, we allow actin polymerization to happen at either end of the cell. The rate of actin polymerization depends on the availability of G-actin but also saturates at large limit of G-actin concentration. This dependence can be described by

(Equation 20) $$J_{\text{actin}}^{f}={\left.J_{a}^{f}\left(\frac{\theta_{c}}{\theta_{c,c}+\theta_{c}}\right)\right|}_{x=x^{t}},J_{actin}^{b}={\left.J_{a}^{b}\left(\frac{\theta_{c}}{\theta_{c,c}+\theta_{c}}\right)\right|}_{x=x^{b,}}$$

where $J_{a}$’s are the coefficient of actin polymerization and $\theta_{c,c}$ is a constant.

The diffusion-reaction-advection equation for the G-actin phase is

(Equation 21) $$\frac{\partial \theta_{c}}{\partial t}+\frac{\partial }{\partial x}\left(\theta_{c}v_{c}\right)=D_{\theta_{c}}\frac{\partial^{2}\theta_{c}}{\partial x^{2}}+\gamma \theta_{n}.$$

The boundary conditions for G-actin are

(Equation 22) $${\left(\theta_{c}v_{c}-D_{\theta_{c}}\frac{\partial \theta_{c}}{\partial x}\right)}_{x^{t}}=\theta_{c}^{f}\frac{dx^{f}}{dt}+J_{\text{actin}}^{f},{\left(\theta_{c}v_{c}-D_{\theta_{c}}\frac{\partial \theta_{c}}{\partial x}\right)}_{x^{b}}=\theta_{c}^{b}\frac{dx^{b}}{dt}-J_{\text{actin}}^{b}.$$

##### Myosin module

Myosin undergoes a continual cycle of activation and inactivation within the cytoplasm. Upon activation, myosin binds to F-actin and moves with F-actin at the velocity $v_{n}$. In contrast, inactivated myosin is transported by the cytosol at the velocity $v_{c}$. Consequently, the mathematical expressions governing the diffusion, convection, and reaction processes for both activated and inactivated myosin are expressed as follows:

(Equation 23) $$\begin{array}{r} \frac{\partial m_{n}}{\partial t}=-\frac{\partial }{\partial x}\left(v_{n}m_{n}-D_{m_{n}}\frac{\partial m_{n}}{\partial x}\right)+k_{\text{on}}m_{c}\theta_{n}-k_{\text{off}}m_{n},\\ \frac{\partial m_{c}}{\partial t}=-\frac{\partial }{\partial x}\left(v_{c}m_{c}-D_{m_{c}}\frac{\partial m_{c}}{\partial x}\right)-k_{on}m_{c}\theta_{n}+k_{\text{off}}m_{n}, \end{array}$$

where $k_{\text{on}}$ and $k_{\text{off}}$ are the rate constant of myosin activation and deactivation, respectively. The boundary conditions are

(Equation 24) $$\begin{array}{l} {\left(v_{n}m_{n}-D_{m_{n}}\frac{\partial m_{n}}{\partial x}\right)}_{x^{b},x^{f}}={\left(m_{n}\frac{dx}{dt}\right)}_{x^{b},x^{f}},\\ {\left(v_{c}m_{c}-D_{m_{c}}\frac{\partial m_{c}}{\partial x}\right)}_{x^{b},x^{f}}={\left(m_{c}\frac{dx}{dt}\right)}_{x^{b},x^{f}}. \end{array}$$

##### Solute module

Confined cells have polarized membrane ion channels, transporters, and pumps that contribute to directional water flux across the cell membrane.^1,5,11,68,85^ Without loss of generality, we will consider electro-neutral solutes. From our prior experience, this approximation has proven effective in modeling osmosis and water flux.^1,2,68,85,89^ Including charged ions, such as sodium, potassium, or chloride, is also possible if necessary.^11,90–92^

The solute flux across passive channels follows the concentration gradient. The fluxes at the front and back of the cell membranes are

(Equation 25) $$J_{p}^{b}=-g^{b}{\left(c_{c}-c_{b}\right)}_{x^{b}},J_{p}^{f}=-g^{f}{\left(c_{c}-c_{f}\right)}_{x^{f}},$$

where $g^{b}$ and $g^{f}$ are membrane permeability to solute. The active fluxes, $J_{c\text{,active}}$, depend on multiple factors, such as the ATP levels or the distribution of pumps. We will also attribute solute flux from ion transporters as active fluxes.^1^ The active fluxes depend on the polarization of such transporters. In the model, the polarization or expression levels of transporters can be determined by experiments. Therefore, we will prescribe $J_{c,\text{active}}^{b}$ and $J_{c,\text{active}}^{f}$ based on the observation of the membrane distribution of solute transporters and pumps. In the model, the difference between $J_{c,\text{active}}^{b}$ and $J_{c,\text{active}}^{f}$ will affect the fluid flow. For this reason, we can set one of the active flux to be zero and only control the other.

The solute diffusion-advection equation and boundary conditions within the cytosol are

(Equation 26) $$\begin{array}{c} \frac{\partial c_{c}}{\partial t}=-\frac{\partial }{\partial x}\left(v_{c}c_{c}-D_{c_{c}}\frac{\partial c_{c}}{\partial x}\right),\\ {\left(v_{c}c_{c}-D_{c_{c}}\frac{\partial c_{c}}{\partial x}\right)}_{x^{b}}={\left.c_{c}\right|}_{x=x^{b}}\frac{dx^{b}}{dt}+J_{p}^{b}+J_{c,active}^{b},\\ {\left(v_{c}c_{c}-D_{c_{c}}\frac{\partial c_{c}}{\partial x}\right)}_{x^{f}}={\left.c_{c}\right|}_{x=x^{f}}\frac{dx^{f}}{dt}+J_{p}^{f}+J_{c,active}^{f}. \end{array}$$

Solute diffusion and advection also happen in the extracellular domains at the back and front of the channel. The corresponding equations and boundary conditions are

(Equation 27) $$\begin{array}{c} \frac{\partial c_{b}}{\partial t}\;=-\frac{\partial }{\partial x}\left(v_{c}c_{b}-D_{c_{b}}\frac{\partial c_{b}}{\partial x}\right),\\ {\left(v_{c}c_{b}-D_{c_{b}}\frac{\partial c_{b}}{\partial x}\right)}_{x^{b}}={\left.c_{b}\right|}_{x=x^{b}}\frac{dx^{b}}{dt}+J_{p}^{b}+J_{c,active}^{b},{\left.c_{b}\right|}_{x=0}=c_{b}^{0},\\ \frac{\partial c_{f}}{\partial t}=-\frac{\partial }{\partial x}\left(v_{c}c_{f}-D_{c_{t}}\frac{\partial c_{f}}{\partial x}\right),\\ {\left(v_{c}c_{f}-D_{c_{f}}\frac{\partial c_{f}}{\partial x}\right)}_{x^{f}}={\left.c_{f}\right|}_{x=x^{f}}\frac{dx^{f}}{dt}-J_{p}^{f}-J_{c,active}^{f},{\left.c_{f}\right|}_{x=L}=c_{f}^{0}, \end{array}$$

where $c_{b}^{0}$ and $c_{f}^{0}$ are solute concentrations at x=0 and x=L, respectively.

##### Numerical methods

The system of equations are solved numerically by using the finite difference method. A time-splitting method is used to split the calculation of each module.^85,89^ The sequence of the splitting is the same as the order of modules presented in the Model Description. The advantage of time-splitting method is that within each module the equations are linear and can be solved without using iteration methods. The fluid module is solved by the implicit method for pressures and velocities. Once the cell membrane velocities are obtained, the positions of the membranes are updated accordingly. The actin, myosin, and solute modules are solved by the explicit method.

The only place where nonlinear still exit is the product of $v_{n}$ and $\theta_{n}$ in the actin module. To resolve this problem, we will transform the F-actin equation into an effective diffusion equation in $\theta_{n}$ and obtain $v_{n}$ through post-processing.^85,88^ Specifically, substituting Equation 16 into Equation 17 gives an effective diffusion equation for F-actin that does not depend on $v_{n}$

(Equation 28) $$\frac{\partial \theta_{n}}{\partial t}=\frac{k_{\sigma_{n}}}{\eta_{it}+\eta_{st}}\frac{\partial^{2}\theta_{n}}{\partial x^{2}}-\frac{\eta_{it}}{\eta_{\text{it}}+\eta_{st}}v_{c}\frac{\partial \theta_{n}}{\partial x}-\frac{k_{\sigma_{a}}}{\eta_{\text{it}}+\eta_{st}}\frac{\partial^{2}m_{n}}{\partial x^{2}}-\gamma \theta_{n}.$$

Equation 16 can be used to solve for $v_{n}\theta_{n}$ in the boundary condition

(Equation 29) $$\theta_{n}v_{n}=\frac{\eta_{it}}{\eta_{\text{it}}+\eta_{st}}v_{c}\theta_{n}-\frac{k_{\sigma_{n}}}{\eta_{\text{it}}+\eta_{st}}\frac{\partial \theta_{n}}{\partial x}+\frac{k_{\sigma_{a}}}{\eta_{\text{it}}+\eta_{st}}\frac{\partial m_{n}}{\partial x}.$$

Combing Equations 19 and 29 gives boundary conditions that does not depend on $v_{n}$

(Equation 30) $$\begin{array}{r} {\left(\frac{\eta_{\text{it}}}{\eta_{\text{it}}+\eta_{st}}v_{c}\theta_{n}-\frac{k_{\sigma_{n}}}{\eta_{\text{it}}+\eta_{st}}\frac{\partial \theta_{n}}{\partial x}\right)}_{x^{f}}=\theta_{n}^{f}\frac{dx^{f}}{dt}-J_{actin}^{f}-{\left(\frac{k_{\sigma_{a}}}{\eta_{it}+\eta_{st}}\frac{\partial m_{n}}{\partial x}\right)}_{x^{f}},\\ {\left(\frac{\eta_{\text{it}}}{\eta_{\text{it}}+\eta_{st}}v_{c}\theta_{n}-\frac{k_{\sigma_{n}}}{\eta_{\text{it}}+\eta_{st}}\frac{\partial \theta_{n}}{\partial x}\right)}_{x^{b}}=\theta_{n}^{b}\frac{dx^{b}}{dt}+J_{\text{actin}}^{b}-{\left(\frac{k_{\sigma_{a}}}{\eta_{\text{it}}+\eta_{st}}\frac{\partial m_{n}}{\partial x}\right)}_{x^{b}}. \end{array}$$

The F-actin velocity can be recovered from Equation 29 at each time step:

(Equation 31) $$v_{n}=\frac{\eta_{\text{it}}}{\eta_{\text{it}}+\eta_{\text{st}}}v_{c}-\frac{1}{\theta_{n}}\frac{k_{\sigma_{n}}}{\left(\eta_{\text{it}}+\eta_{st}\right)}\frac{\partial \theta_{n}}{\partial x}+\frac{1}{\theta_{n}}\frac{k_{\sigma_{a}}}{\left(\eta_{\text{it}}+\eta_{st}\right)}\frac{\partial m_{n}}{\partial x}.$$

With this implementation, the equations for F-actin and G-actin and the boundary conditions can be organized as

(Equation 32) $$\begin{array}{c} \frac{\partial \theta_{n}}{\partial t}=-\frac{\partial }{\partial x}\left(\frac{\eta_{\text{it}}}{\eta_{\text{it}}+\eta_{st}}v_{c}\theta_{n}-\frac{k_{\sigma_{n}}}{\eta_{\text{it}}+\eta_{st}}\frac{\partial \theta_{n}}{\partial x}\right)-\frac{k_{\sigma_{a}}}{\eta_{it}+\eta_{st}}\frac{\partial^{2}m_{n}}{\partial x^{2}}-\gamma \theta_{n},\\ {\left(\frac{\eta_{\text{it}}}{\eta_{\text{it}}+\eta_{st}}v_{c}\theta_{n}-\frac{k_{\sigma_{n}}}{\eta_{\text{it}}+\eta_{st}}\frac{\partial \theta_{n}}{\partial x}\right)}_{x^{f}}=\theta_{n}^{f}\frac{dx^{f}}{dt}-J_{actin}^{f}-{\left(\frac{k_{\sigma_{a}}}{\eta_{\text{it}\;}+\eta_{st}}\frac{\partial m_{n}}{\partial x}\right)}_{x^{f}},\\ {\left(\frac{\eta_{\text{it}}}{\eta_{\text{it}}+\eta_{st}}v_{c}\theta_{n}-\frac{k_{\sigma_{n}}}{\eta_{\text{it}}+\eta_{st}}\frac{\partial \theta_{n}}{\partial x}\right)}_{x^{b}}=\theta_{n}^{b}\frac{dx^{b}}{dt}+J_{actin}^{b}-{\left(\frac{k_{\sigma_{a}}}{\eta_{\text{it}\;}+\eta_{st}}\frac{\partial m_{n}}{\partial x}\right)}_{x^{b}}. \end{array}$$

(Equation 33) $$\begin{array}{c} \frac{\partial \theta_{c}}{\partial t}=-\frac{\partial }{\partial x}\left(v_{c}\theta_{c}-D_{\theta_{c}}\frac{\partial \theta_{c}}{\partial x}\right)+\gamma \theta_{n},\\ {\left(v_{c}\theta_{c}-D_{\theta_{c}}\frac{\partial \theta_{c}}{\partial x}\right)}_{x^{f}}=\theta_{c}^{f}\frac{dx^{f}}{dt}+J_{\text{actin}}^{f},{\left(v_{c}\theta_{c}-D_{\theta_{c}}\frac{\partial \theta_{c}}{\partial x}\right)}_{x^{b}}=\theta_{c}^{b}\frac{dx^{b}}{dt}-J_{\text{actin}}^{b}, \end{array}$$

which are ready to be solved by the explicit method.

##### Model implementation

To model the effect of cell reversal, we define ‘on’ and ‘off’ functions for the parameters that differ before and after cell reversal,

(Equation 34) $$f_{\text{on}}=\frac{1}{1+e^{-\left(t-t_{0}\right)/\tau_{0}}},f_{\text{off}}=\frac{1}{1+e^{\left(t-t_{0}\right)/\tau_{0}}}$$

where $t_{0}$ is the time of reversal and $\tau_{0}$ is a constant. In this model, we let $t_{0}=40\;min$ and $\tau_{0}=50\;s$. In the model, $J_{\text{actin}}^{f}$ and $J_{c\text{,active}}^{f}$ are multiplied by $f_{\text{off}}$, whereas $J_{\text{actin}}^{b}$ and $p_{b}^{0}$ are multiplied by $f_{\text{on}}$. The baseline of actin depolymerization, $\gamma_{0}$, is multiplied by $\left(f_{\text{off}}+0.5\right)$. All the other parameters remain unchanged over the process of computation (Table S1). The initial concentration for F-actin and G-actin is 0.2 mM each, whereas the initial concentration of activated and inactivated myosin is 5 μM each.

### QUANTIFICATION AND STATISTICAL ANALYSIS

Experiments were conducted a minimum of three times, unless stated otherwise. Statistical comparisons were performed using paired or unpaired Student’s *t*-test, one-way analysis of variance (ANOVA) test followed by Tukey’s multiple comparisons test and two-way ANOVA followed by Tukey’s multiple comparisons test. *p* < 0.05 was considered to be statistically significant. GraphPad Prism software versions 9 and 10 were used for statistical analysis.

## Supplementary Material

1

2

3

4

## Figures and Tables

**KEY RESOURCES TABLE:** 

REAGENT or RESOURCE	SOURCE	IDENTIFIER

Antibodies		

Monoclonal Anti-β-Actin antibody produced in mouse	Sigma-Aldrich	Catalog #: A3854; RRID: N/A
Lamin A/C (4C11) Mouse mAb	Cell Signaling	Catalog #: 4777; RRID: AB_10545756
Anti-Myosin IIA, non-muscle antibody produced in rabbit	Sigma-Aldrich	Catalog #: M8064; RRID: AB_260673
Anti-mouse IgG, HRP-linked Antibody	Cell Signaling	Catalog #: 7076S; RRID: AB_330924
Anti-rabbit IgG, HRP-linked Antibody	Cell Signaling	Catalog #: 7074S; RRID: AB_2099233
Mouse IgG_2A_ Isotype Control	R&D Systems	Catalog #: MAB003; RRID: AB_357345
CCR7 Blocking Antibody	R&D Systems	Catalog #: MAB197; RRID: AB_2072803

Chemicals, peptides, and recombinant proteins		

Blebbistatin	BioVision	Catalog #: 2406-1
Y-27632 dihydrochloride	Sigma-Aldrich	Catalog #: Y0503
Latrunculin A	R&D Systems	Catalog #: 3973
Ionomycin calcium salt from Streptomyces conglobatus	Sigma-Aldrich	Catalog #: I0634
BAPTA-AM	Sigma-Aldrich	Catalog #: 196419
EIPA	Enzo Life Sciences	Catalog #: NC1049834
Trichostatin A	Sigma-Aldrich	Catalog #: T8552
Methylstat	Sigma-Aldrich	Catalog #: SML0343

Critical commercial assays		

pHrodo Red AM intracellular pH indicators	Invitrogen	Catalog #: P35372
Fluo-4 Direct^™^ Calcium Assay Kit	Invitrogen	Catalog #: F10471

Experimental models: cell lines		

Wild type HT-1080 fibrosarcoma cells	(Wisniewski et al.)^61^	N/A
MDA-MB-231 breast cancer cells	ATCC	Catalog #: HTB-26
Adipose-Derived Mesenchymal Stem Cells; Normal, Human	ATCC	Catalog #: PCS-500-011
Bone Marrow-Derived Mesenchymal Stem Cells; Normal, Human	ATCC	Catalog #: PCS-500-012
HT-1080 cells expressing LifeAct-GFP and H2B-mChery	(Wisniewski et al.)^61^	N/A
HT-1080 cells expressing optoGEF-RhoA and CAAX-CIBN-GFP	(Yankaskas et al.)^40^	N/A
HT-1080 cells expressing EGFP-KASH2ext	(Mistriotis et al.)^39^	N/A
HT-1080 cells expressing EGFP-KASH2	(Mistriotis et al.)^39^	N/A
HT-1080 cells expressing YFP or TRPM7-YFP	(Yankaskas et al.)^40^	N/A
HT-1080 cells expressing TRPM7-YFP	(Yankaskas et al.)^40^	N/A
HT-1080 scramble control	(Mistriotis et al.)^39^	N/A
*shMYH9* HT-1080 cells	(Mistriotis et al.)^39^	N/A
*shLMNA* HT-1080 cells	(Wisniewski et al.)^61^	N/A
Isogenic breast cancer cell lines- 4T1	(Aslakson et al.), (Dexter et al.), (Bell et al.)^48–50^	N/A
Isogenic breast cancer cell lines- 4T07	(Aslakson et al.), (Dexter et al.), (Bell et al.)^48–50^	N/A
Isogenic breast cancer cell lines- 67NR	(Aslakson et al.), (Dexter et al.), (Bell et al.)^48–50^	N/A
Isogenic breast cancer cell lines- 168 FARN	(Aslakson et al.), (Dexter et al.), (Bell et al.)^48–50^	N/A

Oligonucleotides		

Control siRNA-A	Santa Cruz Biotechnology	Catalog #: sc-37007
si*LMNA* (h)	Santa Cruz Biotechnology	Catalog #: sc-35776
Mycoplasma forward primer: GGGAGCAAACAGGATTAGATACCCT	Invitrogen	(Young et al.)^78^
Mycoplasma reverse primer: TGCACCATCTGTCACTCTGTTAACCTC	Invitrogen	(Young et al.)^78^

Recombinant DNA		

Myosin-IIA-GFP	(Jacobelli et al.)^79^	Addgene Plasmid #38297
pCMV-mCherry-MHC-IIA	(Dulyaninova et al.)^80^	Addgene Plasmid #35687
pGP-CMV-GCaMP6s	(Chen et al.)^81^	Addgene Plasmid #40753

Software and algorithms			

ImageJ	NIH	https://imagej.net/software/fiji/
MATLAB (R2021b)	MathWorks	https://www.mathworks.com/products/matlab.html
Graphpad Prism versions 9 and 10	Graphpad	https://www.graphpad.com/
Biorender	Biorender	https://biorender.com/

## Highlights

- Tight confinement promotes upstream migration of cancerous and non-cancerous cells
- High hydraulic resistance and chemotaxis oppose confinement-induced upstream motility
- Upstream migration entails the polarization and activation of migration machinery components
- Low mechanosensitivity enables metastatic tumor cells to escape high-pressure environments
