# Supplementary material for: Confinement controls the directional cell responses to fluid forces
Source: Cell Rep. Author manuscript; Available in PMC 2024 Oct 22. (PMC11495937; doi:10.1016/j.celrep.2024.114692)
Supplement: 1 [file NIHMS2025364-supplement-1.pdf]

Cell Reports, Volume *43*

Supplemental information

Confinement controls the directional cell responses to fluid forces

Farshad Amiri, Ayuba A. Akinpelu, William C. Keith, Farnaz Hemmati, Ravi S. Vaghasiya, Dylan Bowen, Razan S. Waliagha, Chuanyu Wang, Pengyu Chen, Amit K. Mitra, Yizeng Li, and Panagiotis Mistriotis

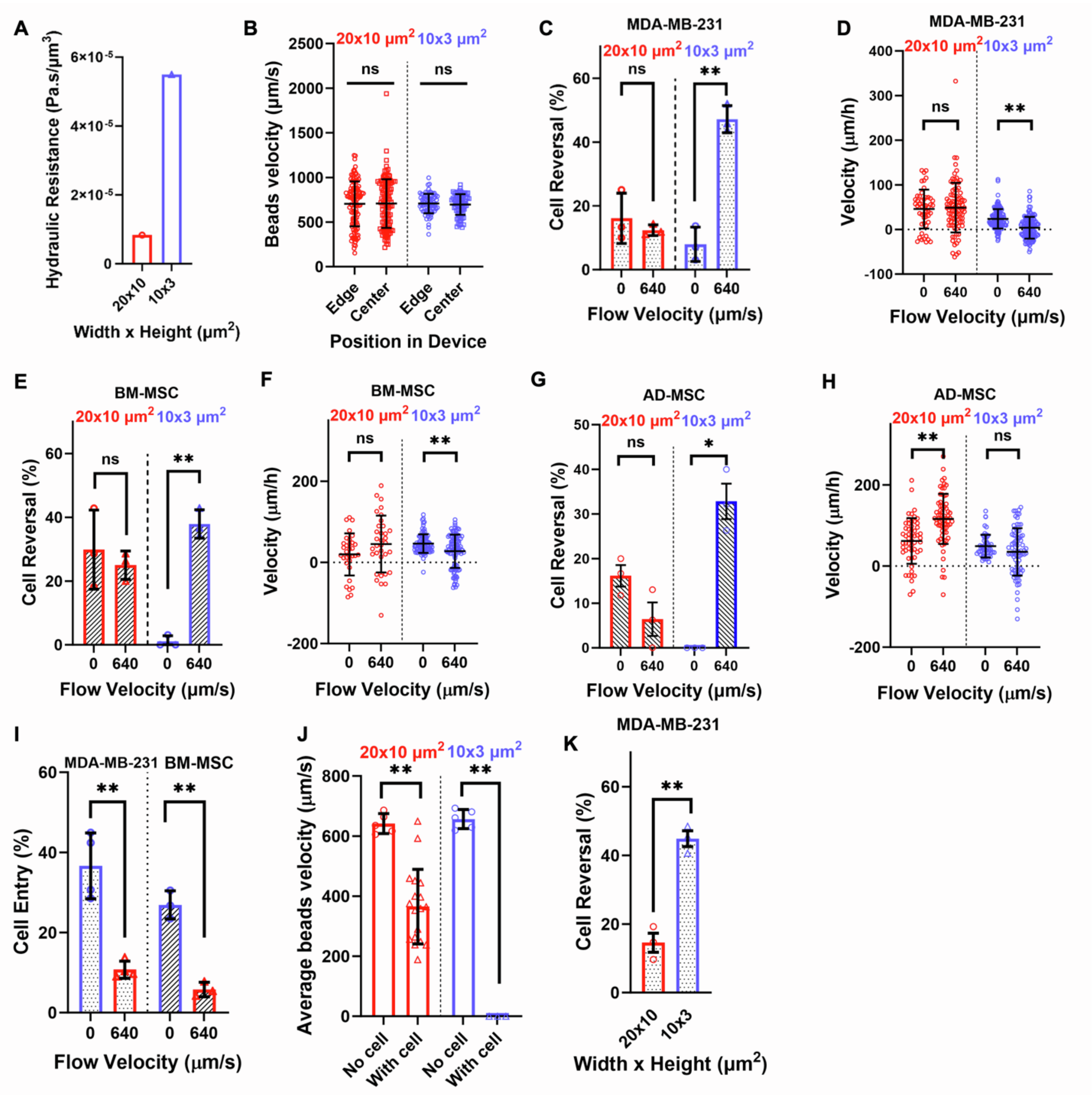

Figure S1. Upstream migration of cancerous and non-cancerous cells in tightly confined microchannels. Related to Figure 1. (A) Total hydraulic resistance of moderately confined (20x10 μm^2) and tightly confined (3x10 μm^2) microfluidic circuits. (B) Beads velocity in moderately and tightly confined microchannels located either at the center or at the edge of cell-free devices. n≥80 beads; 2 independent experiments. (C, E, and G) The percentage of (C) MDA-MB-231 cells and (E) human bone marrow mesenchymal stem cells (BM-MSC) (G) human adipose-derived mesenchymal stem cells (AD-MSC) that reverse their direction in moderately and tightly confined microchannels under static (0 μm/s) and flow (640 μm/s in empty microchannels) conditions. At least 10 cells analyzed per experiment; 3 independent experiments; *p< 0.05, **p< 0.01. (D, F, and H) Cell migration velocity of (D) MDA-MB-231, (F) BM-MSC, and (H) AD-MSC in moderately and tightly confined microchannels under static (0 μm/s) and flow (640 μm/s in empty microchannels) conditions. At least 10 cells analyzed per experiment; 3 independent experiments; **p< 0.01. (I) The percentage of MDA-MB-231 breast cancer cells and BM-MSC that enter tightly confined microchannels under static conditions and following exposure to a 4.8 nN fluid force. At least 143 cells analyzed per experiment; 3 independent experiments; **p< 0.01. (J) Average beads velocity in moderately

and tightly confined devices containing cells. Measurements were conducted in microchannels occupied with HT-1080 cells or devoid of them. $n \geq 4$ cells; 4 independent experiments; **$p < 0.01$. (K) The percentage of MDA-MB-231 cells that reverse their direction in moderately and tightly confined microchannels following exposure to $-\Delta P = 160$ Pa; At least 11 cells analyzed per experiment; 3 independent experiments; **$p < 0.01$. Paired Student's *t*-test (C, E, G, I, and K) and unpaired Student's *t*-test (B, D, F, H, and J) were used for statistical analysis. Values represent mean ± S.D. (B, D, F, H, J) or mean ± S.E.M (C, E, G, I, K).

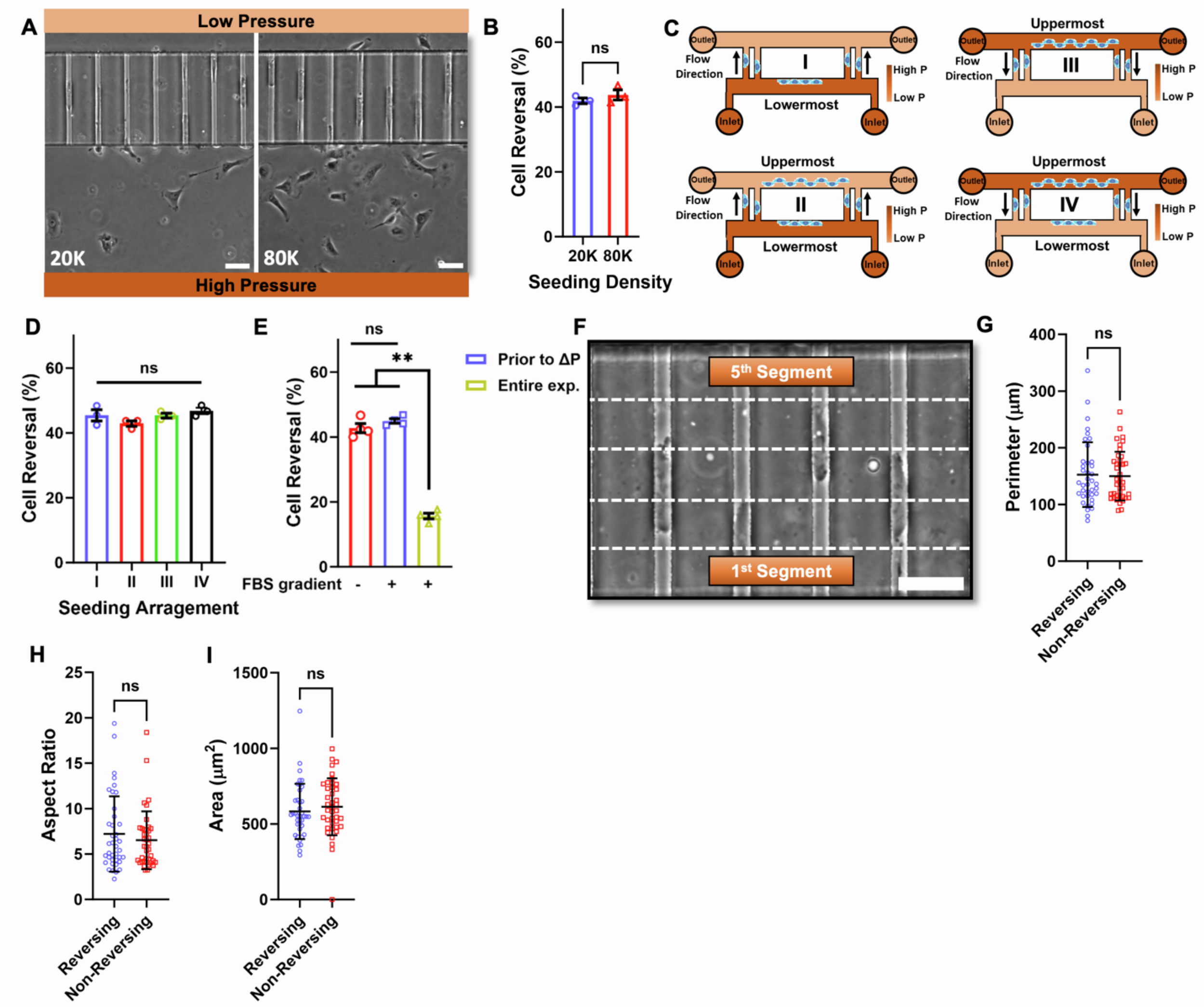

Figure S2. Cell density and precise placement of the cell population in the device do not affect the number of reversing cells. Related to Figure 2. (A) Phase contrast images showing tightly confined devices containing different densities of cells. Scale bar, 50 μm. (B) The percentage of HT-1080 cells that reverse their direction in tightly confined microchannels following exposure to a 4.8 nN fluid force. Cells were seeded in the device at different densities (80k vs 20k). At least 30 cells analyzed per experiment; 3 independent experiments. (C) Schematics showing different cell placements in the device. Cells were seeded in the (I) lowermost channel, (II) uppermost channel, or (III and IV) both channels. In devices III and IV, the direction of flow is opposite compared to devices I and II. (D) The percentage of HT-1080 cells, seeded as shown in (C) that reverse their direction in tightly confined microchannels following exposure to a 4.8 nN fluid force. At least 15 cells analyzed per experiment; 3 independent experiments. (E) The percentage of HT-1080 cells that reverse their direction in tightly confined microchannels following exposure to a 4.8 nN fluid force in the presence or absence of an FBS gradient. Left bar: no FBS gradient; middle bar: use of FBS gradient prior to the application of ΔP; right bar: use of FBS gradient throughout the entire experiment. At least 30 cells analyzed per experiment; 3 independent experiments. $^{**}p < 0.01$. (F) A representative phase-contrast image depicting 200 μm-long microchannels divided into 5 equal segments. Scale bar, 50 μm. (G) Perimeter, (H) aspect ratio, and (I) area of reversing and non-reversing HT-1080 cells immediately after cell exposure to a 4.8 nN of fluid force. n≥39 cells; ≥6 independent experiments. Paired Student's *t*-test (B), unpaired Student's *t*-test (G, H, and I), and one-way ANOVA (D and E) followed by Tukey's multiple comparisons post hoc test were used for statistical analysis. Values represent mean ± S.D. (G, H, I) or mean ± S.E.M (B, D, E).

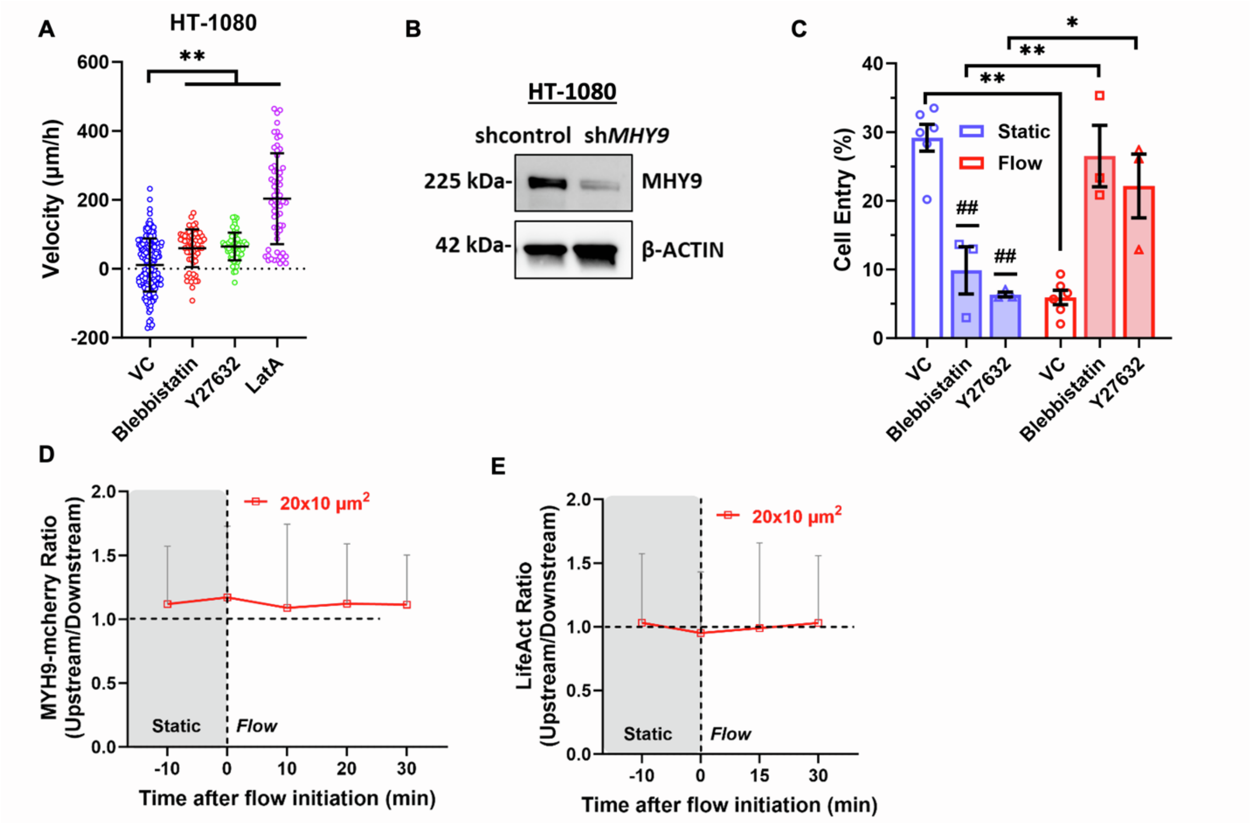

Figure S3. Inhibition of cytoskeletal elements suppresses fluid-force-induced cell reversal. Related to Figure 3. (A) Cell migration velocity of VC-, Blebbistatin-, Y27632- and latrunculin A- (LatA)- treated HT-1080 cells in tightly confined microchannels following exposure to a 4.8 nN fluid force. n=60 cells; 3 independent experiments; $^{**}p < 0.01$. (B) Representative western blot showing the knockdown efficiency of sh*MYH9* in HT-1080 cells. 2 independent experiments. (C) The percentage of vehicle control (VC)-, blebbistatin- and Y27632- treated MDA-MB-231 breast cancer cells that enter tightly confined microchannels under static or flow conditions (640 μm/s in empty microchannels). At least 123 cells analyzed per experiment; 3 independent experiments$^{*}p<0.05$ and $^{**}p< 0.01$ and $^{\#\#}p<0.01$ relative to the static vehicle control. (D) Upstream to downstream ratio of MYH9-GFP signal in moderately confined HT-1080 cells before and after flow initiation (2.5 nN fluid force). n=29 cells; 3 independent experiments. (E) Upstream to downstream ratio of LifeAct-GFP signal in moderately confined HT-1080 cells before and after flow initiation (2.5 nN fluid force). n=30 cells; 3 independent experiments. One-way ANOVA (A, D, E) and two-way ANOVA (C) followed by Tukey's multiple comparisons post hoc test were used for statistical analysis. Values represent mean ± S.D. (A, D, E) or mean ± S.E.M (C).

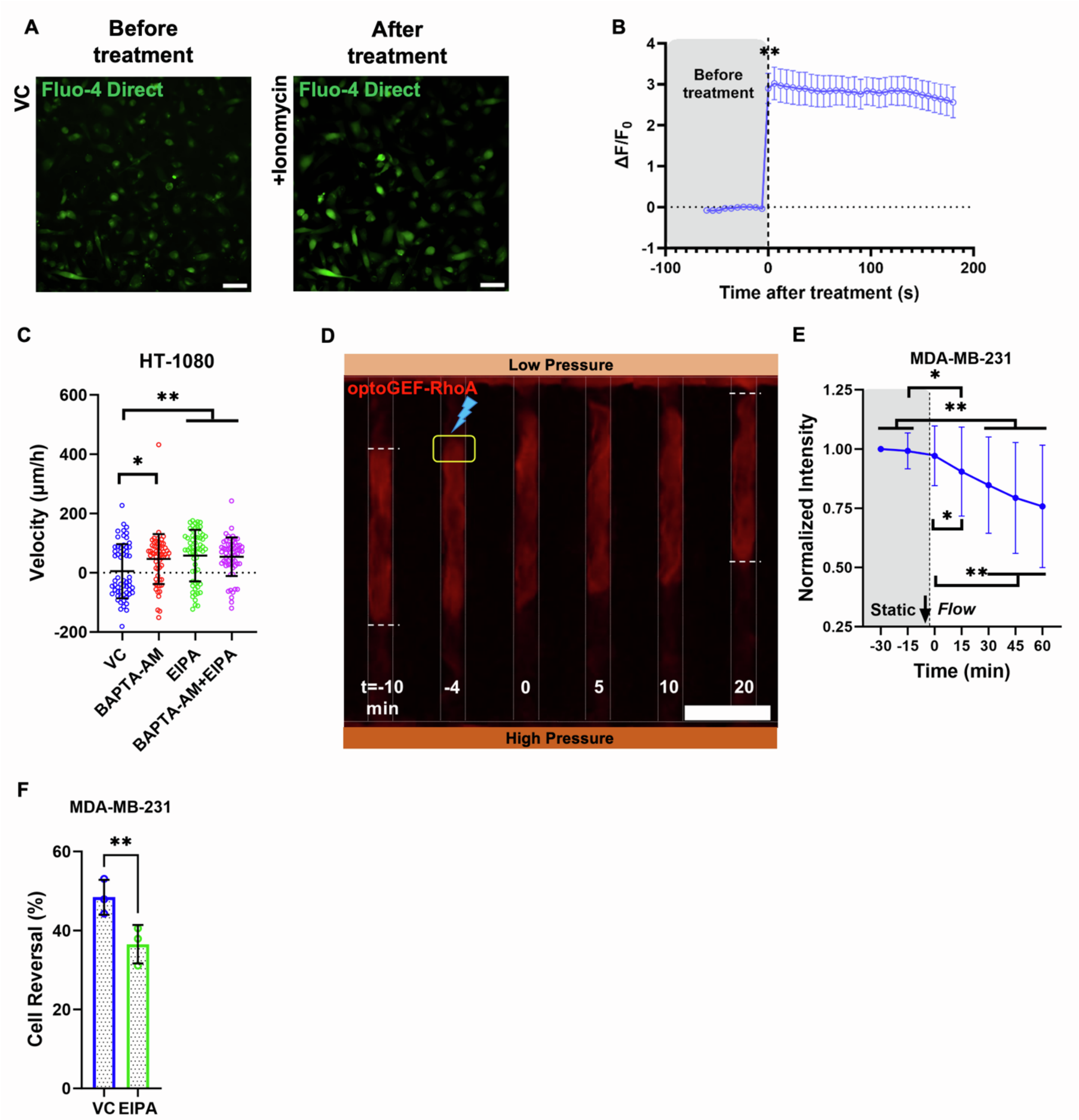

Figure S4. NHE1 and calcium work cooperatively to induce upstream migration in tight confinement. Related to Figure 4. (A) Representative images showing Fluo-4 Direct fluorescence before and after treatment of HT-1080 cells with ionomycin. Scale bar, 50 μm. (B) Quantification of (A). n≥39 cells; 3 independent experiments; **p< 0.01 relative to before treatment. (C) Cell migration velocity of VC-, BAPTA-AM-, EIPA-, and BAPTA-AM+EIPA- treated HT-1080 cells in tightly confined microchannels following exposure to a 4.8 nN fluid force. n=60 cells; 3 independent experiments; *p< 0.05, **p< 0.01. (D) Image sequence of a tightly confined HT-1080 cell expressing only optoGEF-RhoA after initiation of flow (4.8 nN fluid force). The yellow box indicates the region stimulated with blue light. Dotted lines show the position of the cell before and 20 min after its stimulation with light. Scale bar, 30 μm. (E) Normalized fluorescence intensity of pHrodo Red AM in tightly confined MDA-MB-231 cells. The black arrow indicates the initiation of the flow (4.8 nN fluid force). Values are normalized to the intensity of the same cell 30 minutes before flow initiation. n=30 cells; 3 independent experiments; *p< 0.05, **p< 0.01. (F) The percentage of VC-, EIPA-treated MDA-MB-231 cells that reverse their direction in tight confinement under flow conditions (4.8 nN

fluid force); At least 37 cells analyzed per experiment; 3 independent experiments; **$p < 0.01$. Paired Student's *t*-test (F) and one-way ANOVA (B, C, and E) followed by Tukey's multiple comparisons post hoc test were used for statistical analysis. Values represent mean ± S.D. (C, E) or mean ±. S.E.M (B, F).

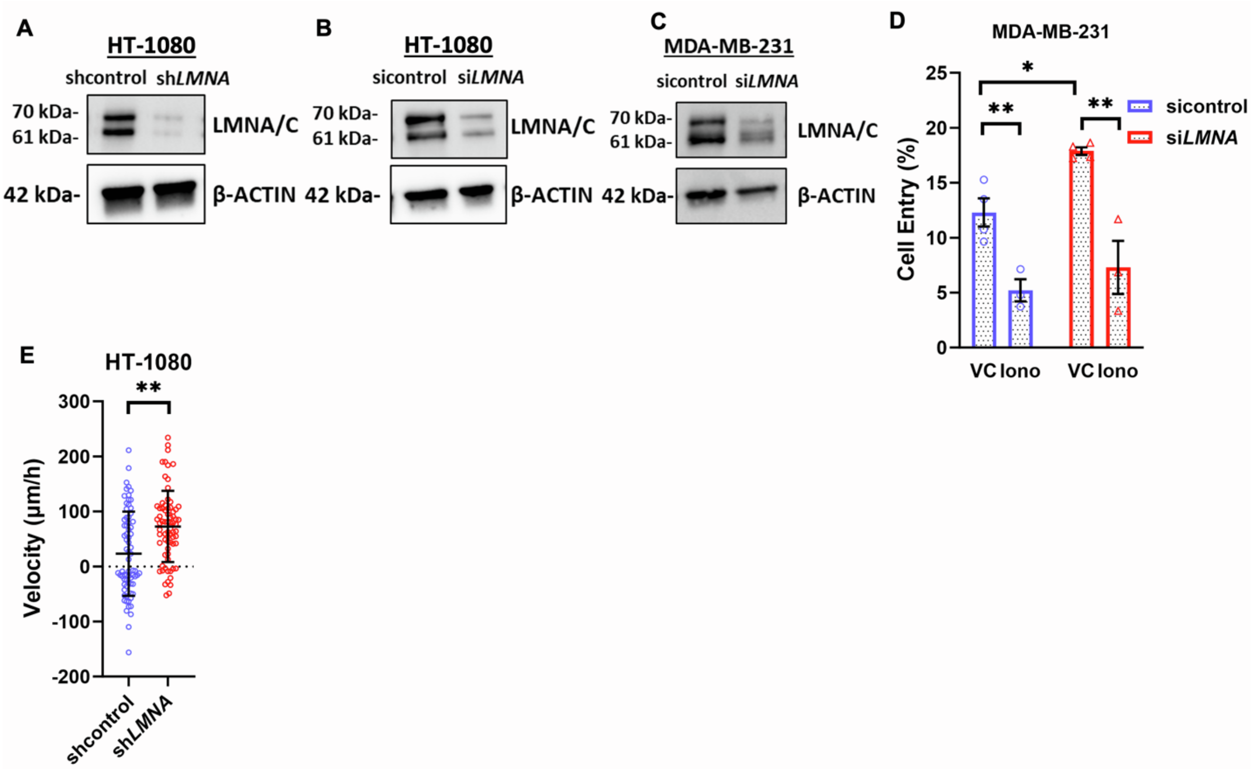

Figure S5. Knockdown of lamn A/C promotes downstream migration. Related to Figure 5. (A-C) Representative western blots showing the knockdown efficiency of (A) sh*LMNA* in HT-1080 cells, (B) si*LMNA* in HT-1080 cells, (C) si*LMNA* in MDA-MB-231 cells. n=2 independent experiments. (D) The percentage of scramble control and lamin A/C KD MDA-MB-231 cells treated with VC or ionomycin that enter tightly confined microchannels under flow condition (640 μm/s in empty microchannels). At least 142 cells analyzed per experiment; 4 independent experiments; *p< 0.05, **p< 0.01. (E) Cell migration velocity of scramble control and lamin A/C KD HT-1080 cells in tightly confined microchannels following exposure to a 4.8 nN fluid force. n=72 cells; 3 independent experiments; **p< 0.01. Unpaired Student's *t*-test (E) and two-way ANOVA (D) followed by Tukey's multiple comparisons post hoc test were used for statistical analysis. Values represent mean ± S.D. (E) or mean ± S.E.M. (D).

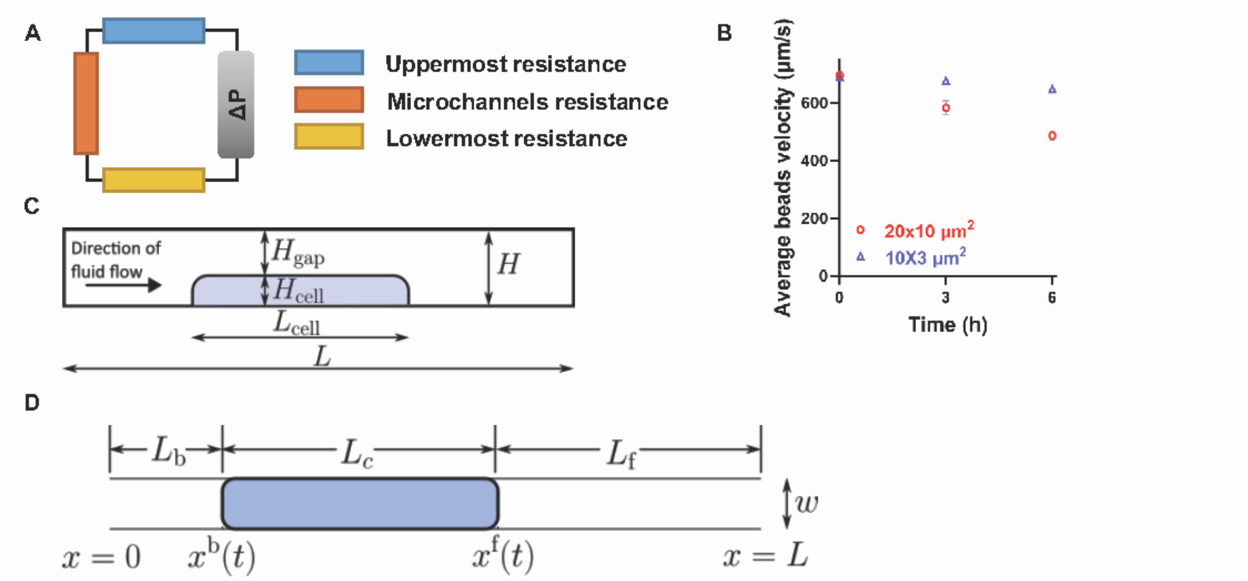

Figure S6. Model for confined migration and estimation of drag forces on confined cells. Related to STAR Methods. (A) Schematic showing the design of the equivalent circuit that allows us to calculate the total resistance of each microfluidic circuit. (B) Average bead velocity in empty moderately and tightly confined channels as a function of time after flow initiation (initial flow velocity of 640 μm/s). Devices contained cells; At least 40 beads analyzed per experiment; 2 independent experiments. (C) Schematic showing the side view of a moderately confined cell. H: the height of the channel; H_{gap}: the height of the empty space above the cell; H_{cell}: the effective height of the cell; L: the length of the channel, L_{cell}: the effective length of the cell; W is the width of the channel, perpendicular into the page (not drawn here). (D) Top view of a cell in a confined channel. The channel has a length of L, a width of W and a height of H. The height direction is perpendicular to the page and is not shown. The scripts 'f' and 'b' denote quantities associated with the front and back of the cell, respectively. The front and back position of the cell are described by $x^{f}(t)$ and $x^{b}(t)$. $L_c(t) = x^{f}(t) - x^{b}(t)$ is the length of the cell.

Table S1: Model parameters, related to STAR Methods.

Parameters	Description	Values	Sources
L (μm)	Channel length	200	Experiments
w (μm)	Channel width	10	Experiments
h (μm)	Channel height	3	Experiments
μ_c (Pa·s)	Cytosol viscosity	2×10^{-3}	Estimated
μ_{f}, μ_{b} (Pa·s)	Extracellular fluid viscosity	1×10^{-3}	From water
η_{it} (Pa·s/μm^2/mM)	Interfacial friction coefficient	1×10^{-3}	[S1, 2]
η_{st} (Pa·s/μm^2/mM)	Strength of focal adhesion	6×10^{2}	[S1, 2]
η_m (Pa·s)	Membrane-cortex viscosity	1	Estimated
k_{ct} (Pa m)	Membrane-cortex stiffness	0.05	Estimated
k_{σ_n} (Pa/mM)	Passive F-actin stress coefficient	8×10^{3}	[S3]
k_{σ_a} (Pa/mM)	Actin myosin contractile stress coefficient	2×10^{4}	[S3]
k_{ad} (Pa·s/μm)	Coefficient of adhesive force	2×10^{4}	[S3]
$\theta_{c,c}$ (μM)	Constant in actin polymerization	0.2	[S3]
J_a^{f}, J_a^{b} (nm mM/s)	Coefficient of actin polymerization	35	[S1-3]
γ_0 (1/s)	Baseline rate of actin depolymerization	3×10^{-3}	Estimated
γ_{a} (1/(Pa·s))	Myosin associated rate of actin depolymerization	1×10^{-5}	Estimated
k_{on} (1/s/mM)	Coefficient for myosin activation	0.4	[S3]
k_{off} (1/s)	Coefficient for myosin deactivation	1.1	[S3]
α_{f}, α_{b} (μm/(Pa s))	Membrane hydraulic conductance	1×10^{-4}	[S2]
D_{c_c}, $D_{c_{\mathrm{f}}}$, $D_{c_{\mathrm{b}}}$ (μm^2/s)	Diffusion coefficient of solute	100	[S1, 2]
D_{θ_c} (μm^2/s)	Diffusion coefficient of G-actin	10	[S3]
D_{m_c} (μm^2/s)	Diffusion coefficient of inactivated myosin	1	[S3]
D_{m_n} (μm^2/s)	Diffusion coefficient of activated myosin	0.1	[S3]

Parameters	Description	Values	Sources
g^{f}, g^{b} (μm/s)	Passive solute permeability	50	[S2]
$J_{c,\mathrm{active}}^{\mathrm{f}}$ (mM μm/s)	Active solute flux	10	Estimated
$J_{c,\mathrm{active}}^{\mathrm{b}}$ (mM μm/s)	Active solute flux	0	Estimated
p_{b}^{0} (Pa)	Hydrostatic pressure at $x = 0$	160	Experiments
p_{f}^{0} (Pa)	Hydrostatic pressure at $x = L$	0	Experiments
c_{b}^{0}, c_{f}^{0} (mM)	Solute concentration at $x = 0, L$	340	[S2]

Supplemental References

1. Li, Y., and Sun, S.X. (2018). Transition from Actin-Driven to Water-Driven Cell Migration Depends on External Hydraulic Resistance. Biophys J 114, 2965-2973. 10.1016/j.bpj.2018.04.045.
2. Li, Y., Yao, L., Mori, Y., and Sun, S.X. (2019). On the energy efficiency of cell migration in diverse physical environments. Proc Natl Acad Sci U S A 116, 23894-23900. 10.1073/pnas.1907625116.
3. Yao, L., Mori, Y., Sun, S.X., and Li, Y. (2023). On the role of myosin-induced actin depolymerization during cell migration. Mol Biol Cell 34, ar62. 10.1091/mbc.E22-10-0494.
